# Time-varying decision boundaries: insights from optimality analysis

**DOI:** 10.3758/s13423-017-1340-6

**Published:** 2017-07-20

**Authors:** Gaurav Malhotra, David S. Leslie, Casimir J. H. Ludwig, Rafal Bogacz

**Affiliations:** 1 School of Experimental Psychology, University of Bristol, 12a Priory Road, Bristol, BS8 1TU UK; 2 Department of Mathematics and Statistics, Lancaster University, Lancaster, UK; 3 MRC Brain Networks Dynamics Unit, University of Oxford, Oxford, UK

**Keywords:** Decision-making, Decreasing bounds, Optimal decisions, Reward rate

## Abstract

The most widely used account of decision-making proposes that people choose between alternatives by accumulating evidence in favor of each alternative until this evidence reaches a decision boundary. It is frequently assumed that this decision boundary stays constant during a decision, depending on the evidence collected but not on time. Recent experimental and theoretical work has challenged this assumption, showing that constant decision boundaries are, in some circumstances, sub-optimal. We introduce a theoretical model that facilitates identification of the optimal decision boundaries under a wide range of conditions. Time-varying optimal decision boundaries for our model are a result only of uncertainty over the difficulty of each trial and do not require decision deadlines or costs associated with collecting evidence, as assumed by previous authors. Furthermore, the shape of optimal decision boundaries depends on the difficulties of different decisions. When some trials are very difficult, optimal boundaries decrease with time, but for tasks that only include a mixture of easy and medium difficulty trials, the optimal boundaries increase or stay constant. We also show how this simple model can be extended to more complex decision-making tasks such as when people have unequal priors or when they can choose to opt out of decisions. The theoretical model presented here provides an important framework to understand how, why, and whether decision boundaries should change over time in experiments on decision-making.

## Introduction

In many environmental settings, people frequently come across decision-making problems where the speed of making decisions trades off with their accuracy. Consider for example the following problem: a financial advisor is employed by a firm to make buy/sell recommendations on their portfolio of assets. All assets seem identical but the value of some assets is stochastically rising while the value of others is falling. For each correct recommendation (advisor recommends buy and the asset turns out to be rising or vice-versa), the advisor receives a fixed commission and for each incorrect recommendation (advisor recommends buy and the asset turns out to be falling or vice-versa) they pay a fixed penalty. In order to make these recommendations, the advisor examines the assets sequentially and observes how the value of each asset develops over time. Each observation takes a finite amount of time and shows whether the value of the asset has gone *up* or *down* over this time. Before recommending whether the firm should buy or sell the asset, the advisor can make as many of these *up*/*down* observations as they like. However, there is an opportunity cost of time as the advisor wants to maximize the commission every month by making as many correct recommendations as possible. How many (*up*/*down*) observations should the advisor make for each asset before giving a recommendation?

### Sequential decision problems

The type of problem described above is at the heart of sequential analysis and has been investigated by researchers from Bernoulli (1713) and Laplace, (1774, 1812) to modern day statisticians (for a review, see Ghosh, 1991). This problem is also directly relevant to the psychology and neuroscience of decision-making. Many decision-making problems, including perceptual decisions (how long to sample sensory information before choosing an option) and foraging problems (how long to forage at the current patch before moving to the next patch), can be described in the form above. The decision-maker has to make a series of choices and the information needed to make these choices is spread out over time. The decision-maker wants to maximize their earnings by attempting as many decisions as possible in the allocated time. Sampling more information (*up*/*down* observations) allows them to be more accurate in their choices, at the expense of the number of decision problems that can be attempted. Therefore the speed of decisions trades off with their accuracy and the decision-maker must solve (i) the *stopping problem*, i.e., decide how much information to sample before indicating their decision, and (ii) the *decision problem*, i.e., which alternative to choose, in such a way that they are able to maximize their earnings.

The stopping problem was given a beautifully simple solution by Wald (1945b), who proposed the following *sequential procedure*: after each sample (*up*/*down* observation), compute the likelihood ratio, *λ*_*n*_, of the samples (*X*_1_,…, *X*_*n*_) and choose the first alternative (buy) if *λ*_*n*_ ≥ *A* and second alternative (sell) if *λ*_*n*_ ≤ *B*, otherwise continue sampling for *n* = 1,2,…, where *A* and *B* are two suitably chosen constants. This procedure was given the name the sequential probability ratio test (SPRT). Wald (1945a, b) and Wald and Wolfowitz (1948) showed that the SPRT is *optimal* in the sense that it can guarantee a required level of accuracy (both Type 1 and Type 2 errors are bounded) with a minimum average sample size (number of *up* /*down* observations made).

This sequential procedure of continuing to sample evidence until a decision variable (likelihood ratio for SPRT) has crossed a fixed threshold also forms the basis for the most widely used psychological account of decision-making. This account consists of a family of models, which are collectively referred to as *sequential sampling models* (Stone, 1960; LaBerge, 1962; Laming, 1968; Link & Heath, 1975; Vickers, 1970; Ratcliff, 1978) and have been applied to a range of decision tasks over the last 50 years (for reviews, see Ratcliff & Smith, 2004; Bogacz, Brown, Moehlis, Holmes, & Cohen, 2006). Like the SPRT, sequential sampling models propose that decision-makers solve the stopping problem by accumulating evidence in favor of each alternative until this evidence crosses a *decision boundary*. Also like the SPRT, the standard sequential sampling account assumes that this decision boundary remains constant during a decision. In fact, (Bogacz et al., 2006) showed that, under certain assumptions, including the assumption that all decisions in a sequence are of the same difficulty, the decision-maker can maximize their reward rate by employing the SPRT and maintaining an appropriately chosen threshold that remains constant within and across trials.^1^ In the above example, this means that if the financial advisor chose the stopping criterion, *stop sampling if you observe three more ups than downs (or vice-versa)*, they stick with this criterion irrespective of whether they have observed ten values of an asset or a hundred.

A number of recent studies have challenged this account from both an empirical and a theoretical perspective, arguing that in many situations decision-makers decrease the decision boundary with time and that it is optimal for them to do so (Drugowitsch, Moreno-Bote, Churchland, Shadlen, & Pouget, 2012; Huang & Rao, 2013; Thura, Cos, Trung, & Cisek, 2014; Moran, 2015). The intuition behind these studies is that instead of determining the decision boundaries based on minimizing the average sample size at a desired level of accuracy (as some formulations of SPRT do), decision-makers may want to maximize the expected reward earned per unit time, i.e., the *reward rate*. Psychological studies and theories of decision-making generally give little consideration to the reward structure of the environment. Participants are assumed to trade-off between accuracy and reaction time in some manner that is consistent with the—typically vague—experimenter instructions (e.g., “try to be as fast and accurate as possible”). Models integrating to a fixed threshold often work well for these situations, giving good accounts for participants’ accuracy and reaction time distributions. However, it has been shown that using models integrating to a fixed threshold leads to sub-optimal reward rate in heterogeneous environments—i.e., when decisions vary in difficulty (Moran, 2015). This leads to the natural question: how should the decision-maker change their decision-boundary with time if their aim was to maximize the reward rate.

### Optimal decision boundaries in sequential decision problems

A number of models have been used to compute the optimal decision boundaries in sequential decision-making. These models differ in (a) how the decision problem is formulated, and (b) whether the decision boundary is assumed to be fixed across trials or vary from one trial to next.

Rapoport and Burkheimer, (1971) modeled the deferred decision-making task (Pitz, Reinhold, & Geller, 1969) where the maximum number of observations were fixed in advance (and known to the observer) and making each observation carried a cost. There was also a fixed cost for incorrect decisions and no cost for correct decisions. Rapoport and Burkheimer used dynamic programming (Bellman, 1957; Pollock, 1964) to compute the policy that minimized the expected loss and found that the optimal boundary collapsed as the number of observations remaining in a trial decreased. Busemeyer and Rapoport, (1988) found that, in such a deferred decision-making task, though people did not appear to follow the optimal policy, they did seem to vary their decision boundary as a function of number of remaining observations.

A similar problem was considered by Frazier and Yu (2007) but instead of assuming that the maximum number of observations was fixed, they assumed that this number was drawn from a known distribution and there was a fixed cost for crossing this stochastic deadline. Like Rapoport and Burkheimer (1971), Frazier and Yu showed that under the pressure of an approaching deadline, the optimal policy is to have a monotonically decreasing decision-boundary and the slope of boundaries increased with the decrease in the mean deadline and an increase in its variability.

Two recent studies analyzed optimal boundaries for a decision-making problem that does not constrain the maximum number of observations. Drugowitsch et al. (2012) considered a very general problem where the difficulty of each decision in a sequence is drawn from a Gaussian or a general symmetric point-wise prior distribution and accumulating evidence comes at a cost for each observation. Using the principle of optimality (Bellman, 1957), Drugowitsch et al. showed that under these conditions, the reward rate is maximized if the decision-maker reduces their decision boundaries with time. Similarly, Huang and Rao (2013) used the framework of partially observed Markov decision processes (POMDP) to show that expected future reward is maximized if the decision-maker reduces the decision boundary with time.

In contrast to the dynamic programming models mentioned above, Deneve (2012) considered an alternative theoretical approach to computing decision boundaries. Instead of assuming that decision boundaries are fixed (though time-dependent) on each trial, Deneve (2012) proposed that the decision boundary is set dynamically on each trial based on an estimate of the trial’s reliability. This reliability is used to get an on-line estimate of the signal-to-noise ratio of the sensory input and update the decision boundary. By simulating the model, Deneve found that decision boundaries maximize the reward rate if they decrease during difficult trials, but increase during easy trials.

### The present analysis

The principal aim of this paper is to identify the minimal conditions needed for time-varying decision boundaries, under the assumption that the decision-maker is trying to maximize the reward rate. We will develop a generic procedure that enables identification of the optimal decision boundaries for *any* discrete, sequential decision problem described at the beginning of this article. In contrast to the problems considered by Rapoport and Burkheimer (1971) and Frazier and Yu (2007), we will show that the pressure of an approaching deadline is *not* essential for a decrease in decision boundaries.

In contrast to Drugowitsch et al. (2012), we do not assume any explicit cost for making observations and show that optimal boundaries may decrease even when making observations carries no explicit cost. Furthermore, unlike the very general setup of Drugowitsch et al. (2012) and Huang and Rao (2013), we make several simplifying assumptions in order to identify how the shape of optimal decision boundaries changes with the constituent difficulties of the task. In particular, in the initial exposition of the model, we restrict the difficulty of each decision to be one of two possible levels (though see the Discussion for a simple extension to more than two difficulties). In doing so, we reveal three key results: (i) we show that optimal boundaries *must* decrease to zero if the mixture of difficulties involves some trials that are uninformative, (ii) the shape of optimal boundaries depends on the inter-trial interval for incorrect decisions but *not* correct decisions (provided the latter is smaller) and (iii) we identified conditions under which the optimal decision boundaries increase (rather than decrease) with time within a trial. In fact, we show that optimal decision boundaries decrease only under a very specific set of conditions. This analysis particularly informs the ongoing debate on whether people and primates decrease their decision boundaries, which has focused on analyzing data from existing studies to infer evidence of decreasing boundaries (e.g., Hawkins, Forstmann, Wagenmakers, Ratcliff, & Brown, 2015; Voskuilen, Ratcliff, & Smith, 2016). The evidence on this point is mixed. Our study suggests that such inconsistent evidence may be due to the way decision difficulties in the experiment are mixed, as well as how the reward structure of the experiment is defined.

Next, we extend this analysis to two situations which are of theoretical and empirical interest: (i) What is the influence of prior beliefs about the different decision alternatives on the shape of the decision boundaries? (ii) What is the optimal decision-making policy when it is possible to *opt-out* of a decision and forego a reward, but be spared the larger penalty associated with an incorrect choice? In each case, we link our results to existing empirical research. When the decision-maker has unequal prior beliefs about the outcome of the decision, our computations show that the optimal decision-maker should dynamically adjust the contribution of prior to each observation during the course of a trial. This is in line with the *dynamic prior model* developed by Hanks, Mazurek, Kiani, Hopp and Shadlen, (2011) but contrasts with the results observed by Summerfield and Koechlin (2010) and Mulder, Wagenmakers, Ratcliff, Boekel, & Forstmann, (2012). Similarly, when it is possible to *opt-out* of a decision, the optimal decision-making policy shows that the decision-maker should choose this option only when decisions involve more than one difficulty (i.e., the decision-maker is uncertain about the difficulty of a decision) and only when the benefit of choosing this option is carefully calibrated.

## A theoretical model for optimal boundaries

### Problem definition

We now describe a Markov decision process to model the stopping problem described at the beginning of this article. We consider the simplest possible case of this problem, where we: (i) restrict the number of choice alternatives to two (buy or sell), (ii) assume that observations are made at discrete (and constant) intervals, (iii) assume that observations consist of binary outcomes (*up* or *down* transitions), and (iv) restrict the difficulty of each decision to one of two possible levels (assets could be rising (or falling) at one of two different rates).

The decision-maker faces repeated decision-making opportunities (trials). On each trial the world is in one of two possible states (asset is rising or falling), but the decision-maker does not know which at the start of the trial. At a series of times steps *t* = 1,2,3,… the decision-maker can choose to *wait* and accumulate evidence (observe if value of asset goes *up* or *down*). Once the decision-maker feels sufficient evidence has been gained, they can choose to *go*, and decide either buy or sell. If the decision is correct (advisor recommends buy and asset is rising or advisor recommends sell and asset is falling), they receive a reward. If the decision is incorrect they receive a penalty. Under both outcomes the decision-maker then faces a delay before starting the next trial. If we assume that the decision-maker will undertake multiple trials, it is reasonable that they will aim to maximize their average reward per unit time. A behavioral policy which achieves the optimal reward per unit time will be found using *average reward dynamic programming* (Howard, 1960; Ross, 1983; Puterman, 2005).

We formalize the task as follows. Let *t* = 1,2,… be discrete points of time during a trial, and let *X* denote the previous evidence accumulated by the decision-maker at those points in time. The decision-maker’s *state* in a trial is given by the pair (*t*, *X*). Note that, in contrast to previous accounts that use dynamic programming to establish optimal decision boundaries (e.g., Drugowitsch et al., 2012; Huang & Rao, 2013), we compute optimal policies directly in terms of evidence and time, rather than (posterior) belief and time. The reasons for doing so are elaborated in the Discussion. In any state, (*t*, *X*), the decision-maker can take one of two *actions*: (i) *wait* and accumulate more evidence (observe asset value goes *up*/*down*), or (ii) *go* and choose the more likely alternative (buy/sell).

If action *wait* is chosen, the decision-maker observes the outcome of a binary random variable, *δX*, where $\mathbb {P}(\delta X=1)=u=1-\mathbb {P}(\delta X=-1)$. The *up-probability*, *u*, depends on the state of the world. We assume throughout that *u* ≥ 0.5 if the true state of the world is rising, and *u* ≤ 0.5 if the true state is falling. The parameter *u* also determines the trial difficulty. When *u* is equal to 0.5, the probability of each outcome is the same (equal probability of asset value going *up*/*down*); consequently, observing an outcome is like flipping an unbiased coin, providing the decision-maker absolutely no evidence about which hypothesis is correct. On the other hand, if *u* is close to 1 or 0 (asset value almost always goes *up*/*down*), observing an outcome provides a large amount of evidence about the correct hypothesis, making the trial easy. After observing *δX* the decision-maker transitions to a new state (*t* + 1, *X* + *δX*), as a result of the progression of time and the accumulation of the new evidence *δX*. Since the decision-maker does not know the state of the world, and consequently does not know *u*, the distribution over the possible successor states (*t* + 1, *X* ± 1) is non-trivial and calculated below. In the most general formulation of the model, an instantaneous cost (or reward) would be obtained on making an observation, but throughout this article we assume that rewards and costs are only obtained when the decision-maker decides to select a go action. Thus, in contrast to some approaches (e.g., Drugowitsch et al., 2012), the cost of making an observation is 0.

If action *go* is chosen then the decision-maker transitions to one of two special states, *C* or *I*, depending on whether the decision made after the *go* action is correct or incorrect. As with transitions under *wait*, the probability that the decision is correct depends in a non-trivial way on the current state, and is calculated below. From the states *C* and *I*, there is no action to take, and the decision-maker transitions to the initial state (*t*, *X*) = (0,0). From state *C* the decision-maker receives a reward *R*_*C*_ and suffers a delay of *D*_*C*_; from state *I* they receive a reward (penalty) of *R*_*I*_ and suffers a delay of *D*_*I*_.

In much of the theoretical literature on sequential sampling models, it is assumed, perhaps implicitly, that the decision-maker knows the difficulty level of a trial. This corresponds to knowledge that the *up-probability* of an observation is *u* = 0.5 + *𝜖* when the true state is rising, and *u* = 0.5 − *𝜖* when the true state is falling. However, in ecologically realistic situations, the decision-maker may not know the difficulty level of the trial in advance. This can be modeled by assuming that the task on a particular trial is chosen from several different difficulties. In the example above, it could be that *up* / *down* observations come from different sources and some sources are noisier than others. To illustrate the simplest conditions resulting in varying decision boundaries, we model the situation where there are only two sources of observations: an easy source with $u \in \mathcal {U}_{e} = \{\frac {1}{2}-\epsilon _{e}, \frac {1}{2}+\epsilon _{e}\}$ and a difficult source with $u \in \mathcal {U}_{d} = \{\frac {1}{2}-\epsilon _{d}, \frac {1}{2}+\epsilon _{d}\}$, where $\epsilon _{e}, \epsilon _{d} \in [0,\frac {1}{2}]$ are the *drifts* of the easy and difficult stimuli, with *𝜖*_*d*_ < *𝜖*_*e*_. Thus, during a difficult trial, *u* is close to 0.5, while for an easy trial *u* is close to 0 or 1. We assume that these two types of tasks can be mixed in any fraction, with $\mathbb {P}(U \in \mathcal {U}_{e})$ the probability that the randomly selected drift corresponds to an easy task in the perceptual environment. For now, we assume that within both of $\mathcal {U}_{e}$ and $\mathcal {U}_{d}$, *u* is equally likely to be above or below 0.5—i.e., there is equal probability of the assets rising and falling. In the section titled “Extensions of the model” below, we will show how our results generalize to the situation of unequal prior beliefs about the state of the world.

**Fig. 1:**
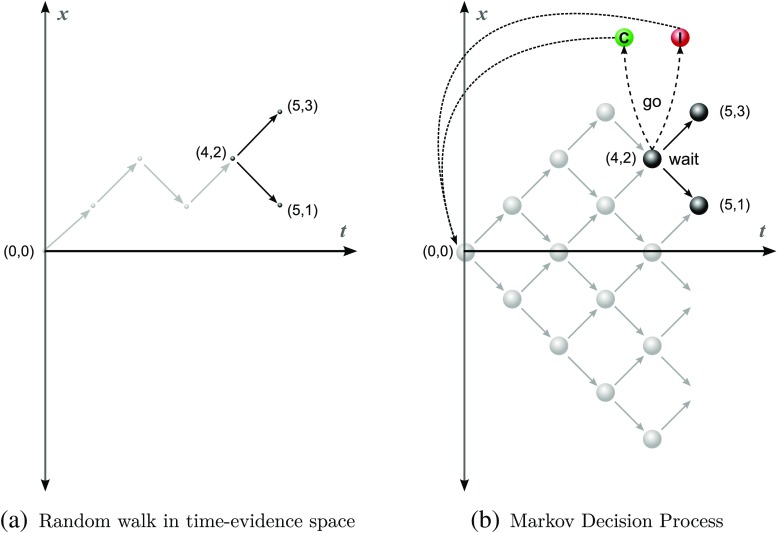
**a** Evidence accumulation as a random walk. *Gray lines* show current trajectory and *black lines* show possible trajectories if the decision-maker chooses to wait. **b** Evidence accumulation and decision-making as a Markov decision process: transitions associated with the action *go* are shown in *dashed lines*, while transitions associated with *wait* are shown in *solid lines*. The rewarded and unrewarded states are shown as C and **I**, respectively (for *Correct* and *Incorrect*)

Figure 1a depicts evidence accumulation as a random walk in two-dimensional space with time along the *x*-axis and the evidence accumulated, *X*_1_,…, *X*_*t*_, based on the series of outcomes, + 1,+1,−1,+1, along the *y*-axis. The figure shows both the current state of the decision-maker at (*t*, *X*_*t*_) = (4,2) and their trajectory in this state-space. In this current state, the decision-maker has two available actions: *wait* or *go*. As long as they choose to *wait* they will make a transition to either (5,3) or (5,1), depending on whether the next *δX* outcome is + 1 or − 1. Figure 1b shows the transition diagram for the stochastic decision process that corresponds to the random walk in Fig. 1a once the *go* action is introduced. Transitions under *go* take the decision-maker to one of the states *C* or *I*, and subsequently back to (0,0) for the next trial.

Our formulation of the decision-making problem has stochastic state transitions, decisions available at each state, and transitions from any state (*t*, *X*) depending only on the current state and the selected action. This is therefore a Markov decision process (MDP) (Howard, 1960; Puterman, 2005), with states (*t*, *x*) and the two dummy states *C* and *I* corresponding to the correct and incorrect choice. A *policy* is a mapping from states (*t*, *x*) of this MDP to *wait*/*go* actions. An optimal policy that maximizes the average reward per unit time in this MDP can be determined by using the policy iteration algorithm (Howard, 1960; Puterman, 2005). A key component of this algorithm is to calculate the average expected reward per unit time for fixed candidate policies. To do so, we must first determine the state-transition probabilities under either action (*wait*/*go*) from each state for a given set of drifts (Eqs. 6 and 7 below). These state-transition probabilities can then be used to compare the *wait* and *go* actions in any given state using the expected reward under each action in that state.

### Computing state-transition probabilities

Computing the transition probabilities is trivial if one knows the *up-probability*, *u*, of the process generating the outcomes: the probability of transitioning from (*t*, *x*) to (*t* + 1, *x* + 1) is *u*, and to (*t* + 1, *x* − 1) is 1 − *u*. However, when each trial is of an unknown level of difficulty, the observed outcomes (*up*/*down*) during a particular decision provide information not only about the correct final choice but also about the difficulty of the current trial. Thus, the current state provides information about the likely next state under a *wait* action, through information about the *up-probability*, *u*. Therefore, the key step in determining the transition probabilities is to *infer* the *up-probability*, *u*, based on the current state and use this to compute the transition probabilities.

As already specified, we model a task that has trials drawn from two difficulties (it is straightforward to generalize to more than two difficulties): easy trials with *u* in the set $\mathcal {U}_{e} = \{\frac {1}{2}-\epsilon _{e}, \frac {1}{2}+\epsilon _{e}\}$ and difficult trials with *u* in the set $\mathcal {U}_{d} = \{\frac {1}{2}-\epsilon _{d}, \frac {1}{2}+\epsilon _{d}\}$ (note that this does not preclude a zero drift condition, *𝜖*_*d*_ = 0). To determine the transition probabilities under the action *wait*, we must marginalize over the set of all possible drifts, $\mathcal {U} = \mathcal {U}_{e} \cup \mathcal {U}_{d}$:

1 $$\begin{array}{@{}rcl@{}} p_{(t,x) \rightarrow (t+1,x+1)}^{wait} \!&=&\! \mathbb{P}(X_{t+1}\,=\,x\,+\,1 | X_{t}\,=\,x)\\ \!&=&\! \sum\limits_{u \in \mathcal{U}} \mathbb{P}(X_{t+1}\,=\,x\,+\,1 | X_{t}\,=\,x,U\,=\,u) \cdot \mathbb{P}(U\,=\,u|X_{t}\,=\,x) \\ p_{(t,x) \rightarrow (t+1,x-1)}^{wait} \!&=&\! 1 - p_{(t,x) \rightarrow (t+1,x+1)}^{wait} \end{array} $$

where *U* is the (unobserved) *up-probability* of the current trial. $\mathbb {P}(X_{t+1}=x+1 | X_{t}=x,U=u)$ is the probability that *δX* = 1 conditional on *X*_*t*_ = *x* and the *up-probability* being *u*; this is simply *u* (the current evidence level *X*_*t*_ is irrelevant when we also condition on *U* = *u*). All that remains is to calculate the term $\mathbb {P}(U=u|X_{t}=x)$.

This posterior probability of *U* = *u* at the current state can be inferred using Bayes’ law:

2 $$ \mathbb{P}(U\,=\,u | X_{t}\,=\,x) \; \,=\, \; \frac{\mathbb{P}(X_{t}\,=\,x | U\,=\,u) \cdot \mathbb{P}(U\,=\,u)} {{\sum}_{\tilde{u}\in\mathcal{U}} \mathbb{P}(X_{t}\,=\,x | U\,=\,\tilde{u}) \cdot \mathbb{P}(U\,=\,\tilde{u})} $$

where $\mathbb {P}(U=u)$ is the prior probability of the *up-probability* being equal to *u*. The likelihood term, $\mathbb {P}(X_{t}=x | U=u)$, can be calculated by summing the probabilities of all paths that would result in state (*t*, *x*). We use the standard observation about random walks that each of the paths that reach (*t*, *x*) contains $\frac {t+x}{2}$ upward transitions and $\frac {t-x}{2}$ downward transitions. Thus, the likelihood is given by the summation over paths of the probability of seeing this number of upward and downward moves:

3 $$ \mathbb{P}(X_{t}\,=\,x | U\,=\,u) \,=\, \sum\limits_{\text{paths}} u^{(t+x)/2}(1\!-u)^{(t-x)/2} \,=\, n_{\text{paths}} u^{(t+x)/2}(1-u)^{(t-x)/2}. $$

Here *n*_paths_ is the number of paths from state (0,0) to state (*t*, *x*), which may depend on the current decision-making policy. Plugging the likelihood into (2) gives

4 $$ \mathbb{P}(U\,=\,u | X_{t}\,=\,x) \; \,=\, \; \frac{ n_{\text{paths}} u^{(t+x)/2}(1\,-\,u)^{(t-x)/2} \mathbb{P}(U\,=\,u)}{{\sum}_{\tilde{u}\in\mathcal{U}} n_{\text{paths}} \tilde{u}^{(t+x)/2}(1\,-\,\tilde{u})^{(t-x)/2}\mathbb{P}(U\,=\,\tilde{u})}. $$

Some paths from (0,0) to (*t*, *x*) would have resulted in a decision to *go* (based on the decision-making policy), and therefore could not actually have resulted in the state (*t*, *x*). Note, however, that the number of paths *n*_paths_ is identical in both numerator and denominator, so can be cancelled.

5 $$ \mathbb{P}(U\,=\,u | X_{t}\,=\,x) \; \,=\, \; \frac{ u^{(t+x)/2}(1-u)^{(t-x)/2} \mathbb{P}(U\,=\,u)}{{\sum}_{\tilde{u}\in\mathcal{U}} \tilde{u}^{(t+x)/2}(1\,-\,\tilde{u})^{(t-x)/2}\mathbb{P}(U\,=\,\tilde{u})}. $$

Using Eq. 1, the transition probabilities under the action *wait* can therefore be summarized as:

6 $$ p_{(t,x) \rightarrow (t+1,x+1)}^{wait} \! = \! \sum\limits_{u \in \mathcal{U}} u \cdot \mathbb{P}(U\,=\,u | X_{t}\,=\,x) \; \,=\, \; 1 - p_{(t,x) \rightarrow (t+1,x-1)}^{wait} $$

where the term $\mathbb {P}(U=u | X_{t}=x)$ is given by Eq. 5. Equation 6 gives the decision-maker the probability of an increase or decrease in evidence in the next time step if they choose to *wait*.

Similarly, we can work out the state-transition probabilities under the action *go*. Under this action, the decision-maker makes a transition to either the correct or incorrect state. The decision-maker will transition to the Correct state if they choose buy and the true state of the world is rising, i.e., true *u* is in $\mathcal {U}_{+} = \{\frac {1}{2}+\epsilon _{e}, \frac {1}{2}+\epsilon _{d}\}$, or if they choose sell and the true state of the world is falling, i.e., true *u* is in $\mathcal {U}_{-} = \{\frac {1}{2}-\epsilon _{e}, \frac {1}{2}-\epsilon _{d}\}$ (assuming *𝜖*_*d*_ > 0; see the end of this section for how to handle *𝜖*_*d*_ = 0).

The decision-maker will choose the more likely alternative–they compare the probability of the unobserved drift *U* coming from the set $\mathcal {U}_{+}$ versus coming from the set $\mathcal {U}_{-}$, given the data observed so far. The decision-maker will respond buy when $\mathbb {P}(U\in \mathcal {U}_{+}|X_{t}=x)>\mathbb {P}(U\in \mathcal {U}_{-}|X_{t}=x)$ and respond sell when $\mathbb {P}(U\in \mathcal {U}_{+}|X_{t}=x)<\mathbb {P}(U\in \mathcal {U}_{-}|X_{t}=x)$. The probability of these decisions being correct is simply the probability of the true states being rising and falling respectively, given the information observed so far. Thus when $\mathbb {P}(U\in \mathcal {U}_{+}|X_{t}=x)>\mathbb {P}(U\in \mathcal {U}_{-}|X_{t}=x)$ the probability of a correct decision is $\mathbb {P}(U\in \mathcal {U}_{+}|X_{t}=x)$, and when $\mathbb {P}(U\in \mathcal {U}_{+}|X_{t}=x)<\mathbb {P}(U\in \mathcal {U}_{-}|X_{t}=x)$ the probability of a correct answer is $\mathbb {P}(U\in \mathcal {U}_{-}|X_{t}=x)$; overall, the probability of being correct is the larger of $\mathbb {P}(U\in \mathcal {U}_{+}|X_{t}=x)$ and $\mathbb {P}(U\in \mathcal {U}_{-}|X_{t}=x)$, meaning that the state transition probabilities for the optimal decision-maker for the action *go* in state (*t*, *x*) are:

7 $$\begin{array}{@{}rcl@{}} p_{(t,x) \rightarrow C}^{go} &=& max \left\{\mathbb{P}(U \in \mathcal{U}_{+} | X_{t}\,=\,x), \mathbb{P}(U \in \mathcal{U}_{-} | X_{t}\,=\,x)\right\}\\ p_{(t,x) \rightarrow I}^{go} &=& 1 - p_{(t,x) \rightarrow C}^{go}. \end{array} $$

Assuming that the prior probability for each state of the world is the same,^2^ i.e., $\mathbb {P}(U \in \mathcal {U}_{+}) = \mathbb {P}(U \in \mathcal {U}_{-})$, the posterior probabilities satisfy $\mathbb {P}(U \in \mathcal {U}_{+} | X_{t}=x) > \mathbb {P}(U \in \mathcal {U}_{-} | X_{t}=x)$ if and only if the likelihoods satisfy $\mathbb {P}(X_{t}=x | U \in \mathcal {U}_{+}) > \mathbb {P}(X_{t}=x | U \in \mathcal {U}_{-})$. In turn, this inequality in the likelihoods holds if and only if *x* > 0. Thus, in this situation of equal prior probabilities, the optimal decision-maker will select buy if *x* > 0 and sell if *x* < 0 so that the transition probability $p_{(t,x) \rightarrow C}^{go}$ is equal to $\mathbb {P}(U \in \mathcal {U}_{+} | X_{t}=x)$ when *x* > 0 and $\mathbb {P}(U \in \mathcal {U}_{-} | X_{t}=x)$ when *x* < 0.

Note that when *𝜖*_*d*_ = 0, a situation which we study below, the sets $\mathcal {U}_{+}$ and $\mathcal {U}_{-}$ intersect, with $\frac {1}{2}$ being a member of both. This corresponds to the difficult trials having an *up-probability* of $\frac 12$ for the true state of the world being either rising and falling. Therefore, in the calculations above, we need to replace $\mathbb {P}(U \in \mathcal {U}_{+} | X_{t}=x)$ in the calculation of the transition probability $p_{(t,x) \rightarrow C}^{go}$ with $\mathbb {P}(U = \frac {1}{2}+\epsilon _{e} | X_{t}=x) + \frac {1}{2} \mathbb {P}(U=\frac {1}{2} | X_{t}=x)$ and $\mathbb {P}(U \in \mathcal {U}_{-} | X_{t}=x)$ with $\mathbb {P}(U = \frac {1}{2}-\epsilon _{e} | X_{t}=x) + \frac {1}{2} \mathbb {P}(U=\frac {1}{2} | X_{t}=x)$.

### Finding optimal actions

In order to find the optimal policy, a dynamic programming procedure called *policy iteration* is used. The remainder of this section provides a sketch of this standard procedure as applied to the model we have constructed. For a more detailed account, the reader is directed towards standard texts on stochastic dynamic programming such as Howard (1960), Ross (1983) and Puterman (2005). The technique searches for the optimal policy amongst the set of all policies by iteratively computing the expected returns for all states for a given policy (step 1) and then improving the policy based on these expected returns (step 2).

#### Step 1: Compute values of states for given *π*

To begin, assume that we have a current policy, *π*, which maps states to actions, and which may not be the optimal policy. Observe that fixing the policy reduces the Markov decision process to a Markov chain. If this Markov chain is allowed to run for a long period of time, it will return an average reward *ρ* ^*π*^ per unit time, independently of the initial state^3^ (Howard, 1960; Ross, 1983). However, the short-run expected earnings of the system will depend on the current state, so that each state, (*t*, *x*), can be associated with a *relative value*, $v_{(t,x)}^{\pi }$, that quantifies the relative advantage of being in state (*t*, *x*) under policy *π*.

Following the standard results of Howard (1960), the relative value of state $v_{(t,x)}^{\pi }$ is the expected value over successor states of the following three components: (i) the instantaneous reward in making the transition, (ii) the relative value of the successor state and (iii) a penalty term equal to the length of delay to make the transition multiplied by the average reward per unit time. From a state (*t*, *x*), under action *wait*, the possible successor states are (*t* + 1, *x* + 1) and (*t* + 1, *x* − 1) with transition probabilities given by Eq. 6; under action *go*, the possible successor states are *C* and *I* with transition probabilities given by Eq. 7; the delay for all of these transitions is one time step, and no instantaneous reward is received. Both *C* and *I* transition directly to (0,0), with reward *R*_*C*_ or *R*_*I*_, and delay *D*_*C*_ or *D*_*I*_ respectively. The general dynamic programming equations reduce to the following

8 $$\begin{array}{@{}rcl@{}} v_{(t,x)}^{\pi} &=& \left\{\begin{array}{ll} p_{(t,x)\to(t+1,x+1)}^{wait} v_{(t+1,x+1)}^{\pi} + p_{(t,x)\to(t+1,x-1)}^{wait} v_{(t+1,x-1)}^{\pi} - \rho^{\pi} &\quad \text{if }\; \pi(t,x)=wait\\ p_{(t,x)\to C}^{go} v_{C}^{\pi} + p_{(t,x)\to I}^{go} v_{I}^{\pi} - \rho^{\pi} &\quad \text{if }\; \pi(t,x)=go \end{array}\right. \\ v_{C}^{\pi} &=& R_{C}+v_{(0,0)}^{\pi} - D_{C}\rho^{\pi}\\ v_{I}^{\pi} &=& R_{I}+v_{(0,0)}^{\pi} - D_{I}\rho^{\pi} \end{array} $$

The unknowns of the system are the relative values $v_{(t,x)}^{\pi }$, $v_{C}^{\pi }$ and $v_{I}^{\pi }$, and the average reward per unit time *ρ* ^*π*^. The system is underconstrained, with one more unknown (*ρ* ^*π*^) than equations. Note also that adding a constant term to all $v^{\pi }_{\cdot }$ terms will produce an alternative solution to the equations. So we identify the solutions by fixing $v_{(0,0)}^{\pi }=0$ and interpreting all other $v^{\pi }_{\cdot }$ terms as being values relative to state (0,0).

#### Step 2: Improve $\pi \rightarrow \pi ^{new}$

So far, we have assumed that the policy, *π*, is arbitrarily chosen. In the second step, we use the relative values of states, determined using Eq. 8, to improve this policy. This improvement can be performed by applying the principle of optimality (Bellman, 1957): in any given state on an optimal trajectory, the optimal action can be selected by finding the action that maximizes the expected return and assuming that an optimal policy will be followed from there on.

When updating the policy, the decision-maker thus selects an action for a state which maximizes the expectation of the immediate reward plus the relative value of the successor state penalized by the opportunity cost, with successor state values and opportunity cost calculated under the incumbent policy *π*. In our model, actions need only be selected in states (*t*, *x*), and we compare the two possible evaluations for $v_{(t,x)}^{\pi }$ in Eq. 8. Therefore the decision-maker sets *π* ^*new*^(*t*, *x*) = *wait* if

9 $$\begin{array}{@{}rcl@{}} p_{(t,x)\to(t+1,x+1)}^{wait} v_{(t+1,x+1)}^{\pi} &+& p_{(t,x)\to(t+1,x-1)}^{wait} v_{(t+1,x-1)}^{\pi}\\ &>& p_{(t,x)\to C}^{go} v_{C}^{\pi} + p_{(t,x)\to I}^{go} v_{I}^{\pi} \end{array} $$

and selects *go* otherwise. Note also that, by Eq. 8 and the identification $v^{\pi }_{(0,0)}=0$, the relative values of the correct and incorrect states satisfy $v_{C}^{\pi } = R_{C}-D_{C}\rho ^{\pi }$ and $v_{I}^{\pi } = R_{I}-D_{I}\rho ^{\pi }$. We therefore see the trade-off between choosing to *wait*, receiving no immediate reward and simply transitioning to a further potentially more profitable state, and choosing *go*, in which there is a probability of receiving a good reward but a delay will be incurred. It will only be sensible to choose *go* if $p_{(t,x)\to C}^{go}$ is sufficiently high, in comparison to the average reward *ρ* ^*π*^ calculated under the current policy *π*. Intuitively, since *ρ* ^*π*^ is the average reward per time step, deciding to *go* and incur the delays requires that the expected return from doing so outweighs the expected opportunity cost $\bar {D}\rho ^{\pi }$ (where $\bar {D}$ is a suitably weighted average of *D*_*C*_ and *D*_*I*_). The new policy can be shown to have a better average reward $\rho ^{\pi ^{new}}$ than *ρ* ^*π*^ (Howard, 1960; Puterman, 2005).

This *policy iteration* procedure can be initialized with an arbitrary policy and iterates over steps 1 and 2 to improve the policy. The procedure stops when the policy *π* ^*new*^ is unchanged from *π*, which occurs after a finite number of iterations, and when it does so it has converged on an optimal policy, *π* ^∗^. This optimal policy determines the action in each state that maximizes the long-run expected average reward per unit time.

For computing the optimal policies shown in this article, we initialized the policy to one that maps all states to the action *go* then performed policy iteration until the algorithm converged. The theory above does not put any constraints on the size of the MDP—the decision-maker can continue to wait an arbitrarily large time before taking the action *go*. However due to computational limitations, we limit the largest value of time in a trial to a fixed value *t*_*max*_ by forcing the decision-maker to make a transition to the incorrect state at *t*_*max*_ + 1; that is, for any *x*, $p_{(t_{max},x) \rightarrow I}^{wait} = 1$. In the policies computed below, we set *t*_*max*_ to a value much larger than the interval of interest (time spent during a trial) and verified that the value of *t*_*max*_ does not affect the policies in the chosen intervals. The code for computing the optimal policies as well as the state-transition probabilities is contained in a Toolbox available on the Open Science Framework (https://osf.io/gmjck/).

## Predicted optimal policies

The theory developed above gives a set of actions (a policy) that optimizes the reward rate. We now use this theory to generate optimal policies for a range of decision problems of the form discussed at the beginning of this article. The transition probabilities and state values computed in Eqs. 6, 7 and 8 are a function of the set of *up-probabilities* ($\mathcal {U}$) and inter-trial delays (*D*_*C*_ and *D*_*I*_). Hence, the predicted policies will also be a function of the given set of *up-probabilities* (i.e., the difficulties) and inter-trial delays. We show below how changing these variables leads to a change in the predicted optimal policies and how these policies correspond to decision boundaries that may or may not vary with time based on the value of these variables.

### Single difficulty

**Fig. 2:**
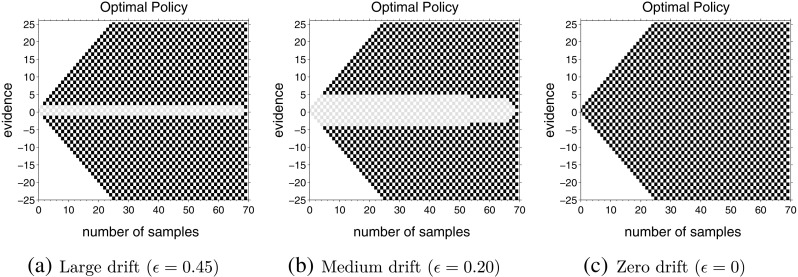
Each panel shows the optimal actions for different points in the state space after convergence of the policy iteration. *Gray squares* indicate that *wait* is the optimal action in that state while *black squares* indicate that *go* is optimal. The inter-trial delays for all three computations were *D*_*C*_ = *D*_*I*_ = 150 and all trials in a task had the same difficulty. The *up-probability* for each decision in the task was drawn, with equal probability from (**a**) *u* ∈{0.05,0.95}, (**b**) *u* ∈{0.30,0.70} and (**c**) *u* = 0.50

We began by computing optimal policies for single difficulty tasks. For the example at the beginning of this article, this means all rising assets go *up* during an observation period with the same probability, $\frac 12 + \epsilon $, and all falling assets go up with the probability $\frac 12 - \epsilon $. Figure 2 shows optimal policies for three different tasks with drifts *𝜖* = 0.45, *𝜖* = 0.20 and *𝜖* = 0, respectively. Panel (a) is a task that consists exclusively of very easy trials, panel (b) consists exclusively of moderately difficult trials and panel (c) consists exclusively of impossible (zero drift) trials. The inter-trial delay in each case was *D*_*C*_ = *D*_*I*_ = 150 (that is, the inter-trial delay was 150 times as long as the delay between two consecutive *up*/*down* observations). The state space shown in Fig. 2 is organized according to number of samples (time) along the horizontal axis and cumulative evidence (*X*_*t*_) along the vertical axis. Each square represents a possible state and the color of the square represents the optimal action for that state, with black squares standing for *go* and light grey squares standing for *wait*. The white squares are combinations of evidence and time that will never occur during a random walk (e.g., (*t*, *x*) = (1,0)) and do not correspond to a state of the MDP.

**Fig. 3:**
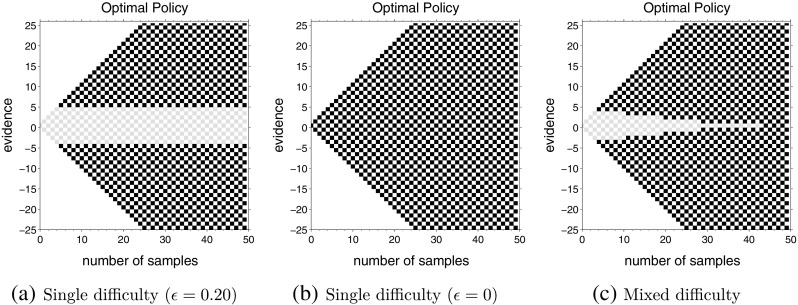
Optimal actions for single and mixed difficulty tasks. The inter-trial intervals used for computing all three policies are *D*_*C*_ = *D*_*I*_ = 150. **a** Single difficulty task with *up-probability* for each decision drawn from *u* ∈{0.30,0.70}; **b** Single difficulty task with $u = \frac {1}{2}$; **c** Mixed difficulty task with *u* ∈{0.30,0.50,0.70}, with both easy and difficult trials equally likely, i.e., $\mathbb {P}(U \in \mathcal {U}_{e}) = \frac {1}{2}$

We can observe from Fig. 2 that, in each case, the optimal policy constitutes a clear decision boundary: the optimal decision is to wait until the cumulative evidence crosses a specific bound. For all values of evidence greater than this bound (for the current point of time), it is optimal to guess the more likely hypothesis. In each panel, the bound is determined by the cumulative evidence, *x*, which was defined above as the difference between number of *up* and *down* observations, |*n*_*u*_ − *n*_*d*_|. Note that, in all three cases, the decision bound stays constant for the majority of time and collapses only as the time approaches the maximum simulated time step, *t*_*max*_. We will discuss the reason for this boundary effect below, but the fact that decision bounds remain fixed prior to this boundary effect shows that it is optimal to have a fixed decision bound if the task difficulty is fixed.

In Fig. 2a and b, the optimal policy dictates that the decision-maker waits to accumulate a criterion level of evidence before choosing one of the options. In contrast, Fig. 2c dictates that the optimal decision-maker should make a decision immediately (the optimal action is to *go* in state (0,0)), without waiting to see any evidence. This makes sense because the *up-probability* for this computation is $u = \frac {1}{2}$; that is, the observed outcomes are completely random without evidence in either direction. So the theory suggests that the decision-maker should not wait to observe any outcomes and choose an option immediately, saving time and thereby increasing the reward rate.

In panels (a) and (b), we can also observe a collapse of the bounds towards the far right of the figure, where the boundary converges to |*n*_*u*_ − *n*_*d*_| = 0. This is a boundary effect and arises because we force the model to make a transition to the incorrect state if a decision is not reached before the very last time step, *t*_*max*_ (in this case, *t*_*max*_ = 70). Increasing *t*_*max*_ moved this boundary effect further to the right, so that it always remained close to the maximum simulated time. In order to prevent confusion and exclude this boundary effect from other effects, all the figures for optimal policies presented below are cropped at *t* = 50: simulations were performed for *t*_*max*_ ≥ 70, but results are displayed until *t* = 50.

In agreement with previous investigations of optimal bounds (Bogacz et al., 2006), computations also showed that the decision boundaries depended *non-monotonically* on the task difficulty, with very high drifts leading to narrow bounds and intermediate drifts leading to wider bounds. Note that the height of the decision boundary is |*n*_*u*_ − *n*_*d*_| = 5 for *𝜖*_*e*_ = 0.20 in Fig. 2b, but decreases on making the task more easy (as in Fig. 2a) as well as more difficult (as in Fig. 2c). Again, this makes intuitive sense: the height of the decision boundary is low when the task consists of very easy trials because each outcome conveys a lot of information about the true state of the world; similarly, decision boundary is low when the task consists of very difficult trials because the decision-maker stands to gain more by making decisions quickly than observing very noisy stimuli.

### Mixed difficulties

Next, we computed the optimal policies when a task contained mixture of two types of decisions with different difficulties. For the example at the beginning of this article, this means some rising assets go *up* during an observation period with the probability $\frac 12 + \epsilon _{e}$ while others go *up* with the probability $\frac 12 + \epsilon _{d}$. Similarly, some falling assets go up with the probability $\frac 12 - \epsilon _{e}$ while others go up with probability $\frac 12 - \epsilon _{d}$. Figure 3 shows the optimal policy for two single difficulty tasks, as well as a mixed difficulty task (Fig. 3c), in which trials can be either easy or difficult with equal probability ($\mathbb {P}(U \in \mathcal {U}_{e}) = \frac {1}{2}$). The drift of the easy task is *𝜖*_*e*_ = 0.20 and the difficult task is *𝜖*_*d*_ = 0.

The optimal policies for the single difficulty tasks (Fig. 3a and b) are akin to the optimal policies in Fig. 2. The most interesting aspect of the results is the optimal policy for mixed difficulty condition (Fig. 3c). In contrast to single difficulty conditions, we see that the decision boundary under this condition is time-dependent. Bounds are wide at the start of the trial (|*n*_*u*_ − *n*_*d*_| = 4) and narrow down as time goes on (reaching |*n*_*u*_ − *n*_*d*_| = 0 at *t* = 44). In other words, the theory suggests that the optimal decision-maker should start the trial by accumulating information and trying to be accurate. But as time goes on, they should decrease their accuracy and guess. In fact, one can analytically show that the decision boundaries will eventually collapse to |*n*_*u*_ − *n*_*d*_| = 0 if there is a non-zero probability that one of the tasks in the mixture has zero drift (*𝜖*_*d*_ = 0) (see Appendix A).

**Fig. 4:**
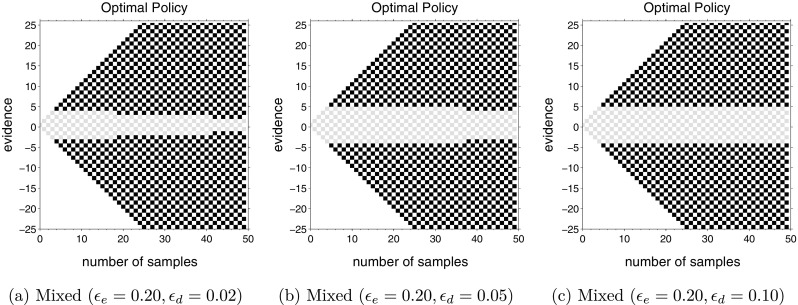
Optimal actions for mixed difficulty tasks with different difficulty levels. Each panel shows mixed difficulty task with *up-probability* for each decision drawn from (**a**) *u* ∈{0.30,0.48,0.52,0.70}, (**b**) *u* ∈{0.30,0.45,0.55,0.70}, and (**c**) *u* ∈{0.30,0.40,0.60,0.70} with equal probability. All other parameters remain the same as in computations shown in Fig. 3 above

We also explored cases with a mixture of decision difficulties, but where the difficult decisions had a positive drift (*𝜖*_*d*_ > 0). Figure 4 shows optimal policies for the same parameters as Fig. 3c, except the drift of the difficult decisions has been changed to *𝜖*_*d*_ = 0.02, 0.05, and 0.10, respectively. The drift for the easy decisions remained *𝜖*_*e*_ = 0.20. Bounds still decrease with time when *𝜖*_*d*_ = 0.02 and 0.05 but the amount of decrease becomes negligible very rapidly. In fact, when *𝜖*_*d*_ = 0.10, the optimal policy (at least during the first 50 time-steps) is exactly the same as the single difficulty task, with *𝜖* = 0.20 (compare with Fig. 3a). We explored this result using several different values of inter-trial intervals and consistently found that decision boundaries show an appreciable collapse for only a small range of decision difficulties and, in particular, when one type of decision is extremely difficult or impossible.

An intuitive explanation for collapsing bounds in Figs. 3c and 4a, b could be as follows: the large drift (easier) task (Fig. 3a) has wider bounds than the small drift task (Fig. 3b); with the passage of time, there is a gradual increase in the probability that the current trial pertains to the difficult task if the boundary has not yet been reached. Hence, it would make sense to start with wider bounds and gradually narrow them to the bounds for the more difficult task as one becomes more certain that the current trial is difficult. If this explanation is true, then bounds should decrease for a mixture of difficulties only under the condition that the easier task has wider bounds than the more difficult task.

### Increasing bounds

**Fig. 5:**
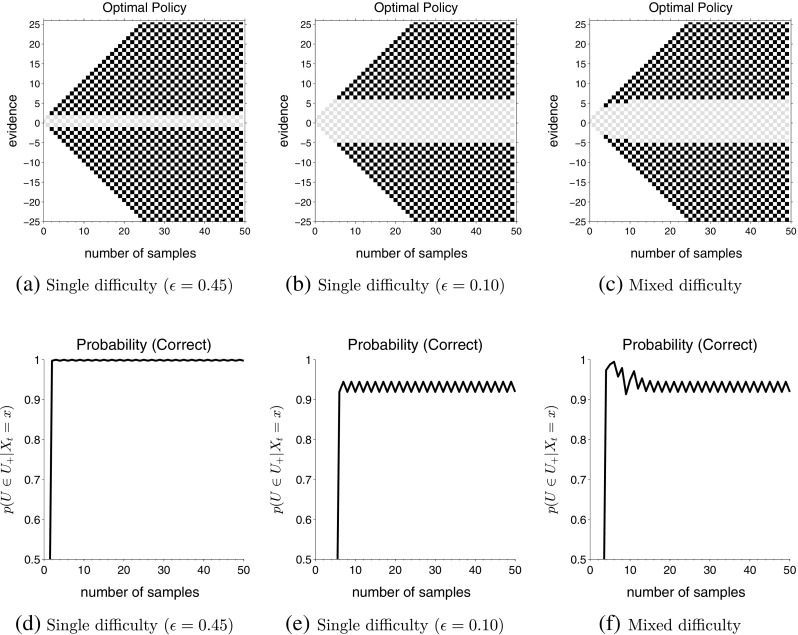
Optimal actions for a mixture of difficulties when the easy task has narrower bounds than the difficult task. The inter-trial delays for all three computations are *D*_*C*_ = *D*_*I*_ = 150. Panels (**a**) and (**b**) show optimal policies for single difficulty tasks with *up-probability* of each decision chosen from *u* ∈{0.05,0.95} and *u* ∈{0.40,0.60}, respectively. Panel (**c**) shows optimal policy in mixed difficulty task with *up-probability* chosen from *u* ∈{0.05,0.40,0.60,0.95} and $\mathbb {P}(U \in \mathcal {U}_{e}) = \mathbb {P}(U \in \mathcal {U}_{d}) = \frac {1}{2}$. Panels (**d**–**f**) show the change in posterior probabilities $\mathbb {P}(U \in \mathcal {U}_{+} | X_{t}=x)$ with time at the upper decision boundary for conditions (**a**–**c**), respectively

The next set of computations investigated what happens to decision boundaries for mixed difficulty task when the easier task has narrower bounds than the more difficult task. Like Fig. 3, Fig. 5 shows optimal policies for two single difficulty tasks and a mixed difficulty task that combines these two difficulties. However, in this case, the two single difficulty tasks are selected so that the bounds for the large drift (easy) task (Fig. 5a) are narrower than the small drift (difficult) task (Fig. 5b), reversing the pattern used in the set of tasks for Fig. 3. Figure 5c shows the optimal actions in a task where these two difficulty levels are equally likely. In contrast to Fig. 3c, the optimal bounds for this mixture are narrower at the beginning, with |*n*_*u*_ − *n*_*d*_| = 4 and then get wider, reaching |*n*_*u*_ − *n*_*d*_| = 6 and then stay constant. Thus, the theory predicts that inter-mixing difficulties does not necessarily lead to monotonically collapsing bounds.

In order to get an insight into why the optimal boundary increases with time in this case, we computed the posterior probability of making the correct decision at the optimal boundary. An examination of this probability showed that although the optimal boundary is lower for the easy task (Fig. 5a) than for the difficult task (Fig. 5b), the posterior $\mathbb {P}(U \in \mathcal {U}_{+} | X_{t}=x)$ at which the choice should be made is higher for the easy task (Fig. 5d) than for the difficult task (Fig. 5e). For the mixed difficulty task, although the optimal boundary increases with time (Fig. 5c), the probability of making a correct choice decreases with time (Fig. 5f).^4^ This happens because the posterior probability of the current trial being difficult increases with time. This fits well with the intuitive explanation of time-varying decision boundaries given for collapsing bounds. At the start of the trial, the decision-maker does not know whether the trial is easy or difficult and starts with a decision boundary somewhere between those for easy and difficult single difficulty tasks. As time progresses and a decision boundary is not reached, the probability of the trial being difficult increases and the decision boundaries approach the boundaries for the difficult task. Since the decision boundaries for the difficult task (*𝜖*_*d*_ = 0.10) are wider than the easy task (*𝜖*_*e*_ = 0.45) in Fig. 5, this means that the decision boundaries increase with time during the mixed difficulty task.

We computed the optimal policies for a variety of mixing difficulties and found that bounds increase, decrease or remain constant in a pattern that is consistent with this intuitive explanation: when the task with smaller drift (the more difficult task) has narrower bounds than the task with larger drift (as in Fig. 3), mixing the two tasks leads to either constant bounds in-between the two bounds, or to monotonically decreasing bounds that asymptote towards the narrower of the two bounds. In contrast, when the task with the smaller drift has wider bounds than the task with larger drift (as in Fig. 5), mixing the two tasks leads to either constant bounds in-between the two bounds or bounds that increase and then asymptote towards the wider of the two bounds.

### Effect of inter-trial intervals

**Fig. 6:**
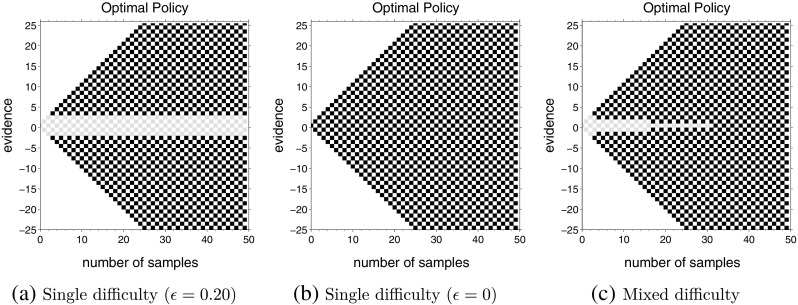
Optimal actions for single and mixed difficulty tasks when inter-trial intervals are reduced to *D*_*C*_ = *D*_*I*_ = 50. All other parameters are the same as Fig. 3

The computations so far have focused on how the difficulty level (drift) affects the optimal policy. Therefore, all computations shown so far used the same inter-trial intervals (*D*_*C*_ = *D*_*I*_ = 150) but varied the drift. However, our conclusions about policies in single and mixed difficulty conditions are not restricted to a particular choice of inter-trial delay. Figure 6, for example, shows how optimal policy changes when this delay is changed. To generate these policies, the inter-trial delay was decreased to *D*_*C*_ = *D*_*I*_ = 50. All other parameters were the same as those used for computing policies in Fig. 3.

A comparison of Figs. 3a and 6a, which have the same drifts but different inter-trial intervals, shows that the optimal bounds decrease from |*n*_*u*_ − *n*_*d*_| = 5 when the inter-trial delay is 150 to |*n*_*u*_ − *n*_*d*_| = 3 inter-trial delay is reduced to 50. Intuitively, this is because decreasing the inter-trial intervals alters the balance between *wait* ing and *go* ing (Eq. 8), making *go* ing more favorable for certain states. When the inter-trial interval decreases, an error leads to a comparatively smaller drop in the reward rate as the decision-maker quickly moves on to the next reward opportunity. Therefore, the decision-maker can increase their reward rate by lowering the height of the boundary to *go*. A comparison of Figs. 3c and 6c shows that a similar result holds for the mixed difficulty condition: decision boundaries still decrease with time, but the boundary becomes lower when inter-trial delay is decreased.

Thus far, we have also assumed that the inter-trial intervals for correct and error responses (*D*_*C*_ and *D*_*I*_, respectively) are the same. In the next set of computations, we investigated the shape of decision boundaries when making an error carried an additional time-penalty, *D*_*p*_, so that the delay is *D*_*C*_ after correct response and *D*_*C*_ + *D*_*p*_ after errors.

An unintuitive result from previous research (Bogacz et al., 2006) is that different combinations of *D*_*C*_ and *D*_*p*_ that have the same sum (*D*_*C*_ + *D*_*p*_), lead to the same boundary. So, for example, the optimal boundaries are the same when both correct and incorrect decisions lead to an equal delay of 150 time steps as when the correct decisions lead to a delay of 75 time steps but the incorrect decisions lead to an additional 75 time steps.

**Fig. 7:**
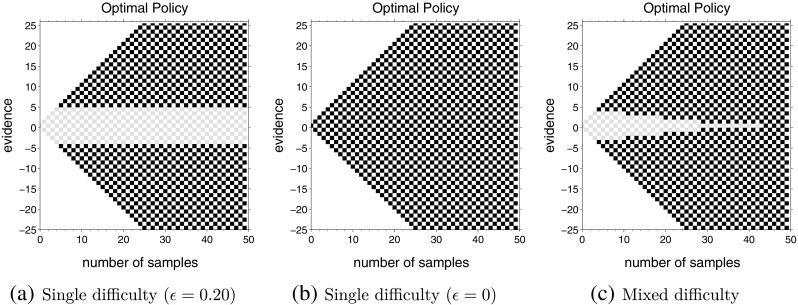
Optimal actions remain the same if *D*_*C*_ + *D*_*p*_ remain the same. Each panel shows the optimal policy for *up-probability* drawn from the same set as Fig. 3, but for an inter-trial delay of *D*_*C*_ = 75 for correct guesses and *D*_*I*_ = 150 for errors

Results for computations shown in Fig. 7 indicate that this property generalizes to the case of mixed difficulties. The optimal policy for single and mixed difficulty tasks in this figure are obtained for *up-probability* drawn from the same set as in Fig. 3, but with delays of *D*_*C*_ = 75 and *D*_*I*_ = *D*_*C*_ + *D*_*p*_ = 150. Comparing Figs. 3 and 7, one can see that changing the delays has not affected the decision boundaries at all. This is because even though *D*_*p*_ = 75 for Fig. 7, *D*_*C*_ + *D*_*p*_ was the same as Fig. 3. Moreover, not only are the boundaries the same for the single difficulty conditions (as previously shown), they are also the same for the corresponding mixed difficulty conditions.

## Extensions of the model

The theoretical model outlined above considers a simplified decision-making task, where the decision-maker must choose from two equally likely options. We now show how the above theory generalizes to situations where: (a) the world is more likely to be in one state than the other (e.g., assets are more likely to be falling than rising), and (b) the decision-maker can give up on a decision that appears too difficult (make no buy or sell recommendation on an asset). In each case, the normative model illuminates how the sequential sampling models should be adapted for these situations.

### Prior beliefs about the world

First, consider the assumption that both states of the world are equally likely. A key question in perceptual decision-making is how decision-makers combine this prior belief with samples (cues) collected during the trial. The effect of prior information on decision-making can be *static*, i.e., remain constant during the course of a decision, or *dynamic*, i.e., change as the decision-maker samples more information. Correspondingly, sequential sampling models can accommodate the effect of prior in either the starting point if the effect of prior is static or in the drift or threshold if the effect is dynamic (Ashby, 1983; Ratcliff, 1985; Diederich & Busemeyer, 2006; Hanks et al., 2011).

Experiments with humans and animals investigating whether the effect of prior beliefs is constant or changes with time, have led to mixed results. A number of recent experiments have shown that shifting the starting point is more parsimonious with the accuracy and reaction time of participants (Summerfield & Koechlin, 2010; Mulder et al., 2012). However, these experiments only consider a single task difficulty. In contrast, when (Hanks et al., 2011) considered a task with a mixture of difficulties, they found that data from the experiment can be better fit by a time-dependent prior model. Instead of assuming that the effect of a prior bias is a shift in starting point, this model assumes that the prior dynamically modifies the decision variable—i.e., the decision variable at any point is the sum of the drift and a *dynamic bias signal* that is a function of the prior and increases monotonically with time.

We examined this question from a normative perspective—should the effect of a prior belief be time-dependent if the decision-maker wanted to maximize reward rate? (Edwards, 1965) has shown that when the reliability of the task is known and constant, the optimal strategy is to shift the starting point. More recently, (Huang, Hanks, Shadlen, Friesen, & Rao, 2012) argued that instead of modeling the data in terms of a sequential sampling model with adjustment to starting point or drift, the decisions in experiments such as (Hanks et al. 2011) can be adequately described by a POMDP model that assumed priors to be distributed according to a (piecewise) Normal distribution and maximized the reward. We will now show that a normative model that maximizes the reward rate, such as the model proposed by Huang et al. (2012), is in fact, consistent with sequential sampling models. Whether the effect of prior in such a model is time-dependent or static depends on the mixture of difficulties. As observed by Hanks et al. (2011), in mixed difficulty situations, the passage of time itself contains information about the reliability of stimuli: the longer the trial has gone on, the more unreliable the source of stimuli is likely to be and decision-makers should increasingly trust their prior beliefs.

For the MDP shown in Fig. 1b, in any state (*t*, *x*), the effect of having biased prior beliefs is to alter the transition probabilities for *wait* as well as *go* actions. We can see that changing the prior in Eq. 5 will affect the posterior probability $\mathbb {P}(U=u | X_{t}=x)$, which, in turn, affects the transition probability $p_{(t,x) \rightarrow (t+1,x+1)}^{wait}$ in Eq. 6. Similarly, a change in the prior probabilities changes the posteriors $\mathbb {P}(U \in \mathcal {U}_{+} | X_{t}=x)$ and $\mathbb {P}(U \in \mathcal {U}_{-} | X_{t}=x)$ in Eq. 7, in turn changing the transition probability $p_{(t,x) \rightarrow C}^{go}$. We argued above that when priors are equal, $\mathbb {P}(U \in \mathcal {U}_{+}) = \mathbb {P}(U \in \mathcal {U}_{-})$, the optimal decision-maker should recommend buy or sell based solely on the likelihoods: i.e., buy whenever *x* > 0 and recommend sell whenever *x* < 0. This will no longer be the case when the priors are unequal. In this case, the transition probabilities under the action *go* will be given by the more general formulation in Eq. 7, i.e., buy whenever the posterior probability for rising is larger than falling ($\mathbb {P}(U \in \mathcal {U}_{+} | X_{t}=x) > \mathbb {P}(U \in \mathcal {U}_{-} | X_{t}=x)$) and sell otherwise.

**Fig. 8:**
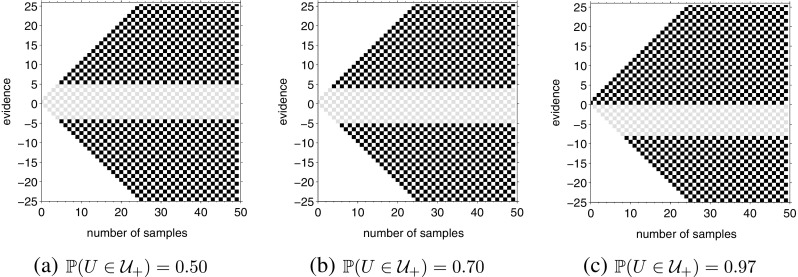
Change in optimal policy during single difficulty tasks with increasingly biased prior beliefs. **a** $\mathbb {P}(U \in \mathcal {U}_{+}) = 0.50$; **b** $\mathbb {P}(U \in \mathcal {U}_{+}) = 0.70$; **c** $\mathbb {P}(U \in \mathcal {U}_{+}) = 0.97$. For all three computations, *up-probability* is drawn from *u* ∈{0.30,0.70} and the inter-trial intervals are *D*_*C*_ = *D*_*I*_ = 150

Figure 8 shows how the optimal policy changes when the prior belief changes from both states of the world being equally probable (assets are equally likely to rise and fall) to one state being more probable than the other (assets are more likely to rise than fall). All policies in Fig. 8 are for single difficulty tasks where the difficulty (drift) is fixed and known ahead of time.

We can observe that a bias in the prior beliefs shifts the optimal boundaries: when the prior probabilities of the two states of the world were the same ($\mathbb {P}(U \in \mathcal {U}_{+}) = \mathbb {P}(U \in \mathcal {U}_{-})$), the height of the boundary for choosing each alternative was |*n*_*u*_ − *n*_*d*_| = 5 (Fig. 8a). Increasing the prior probability of the world being in the first state to $\mathbb {P}(U \in \mathcal {U}_{+}) = 0.70$ reduces the height of the boundary for choosing the first alternative to (*n*_*u*_ − *n*_*d*_) = 4, while it increases the height of the boundary for choosing the other alternative to (*n*_*u*_ − *n*_*d*_) = −6 (Fig. 8b). Thus, the optimal decision-maker will make decisions more quickly for trials where the true state of the world matches the prior but more slowly when the true state and the prior mismatch. Furthermore, note that the increase in boundary in one direction exactly matches the decrease in boundary in the other direction, so that the change in boundaries is equivalent to a shift in the starting point, as proposed by Edwards (1965). Increasing the bias in prior further (Fig. 8c) increased this shift in boundaries, with the height of the boundary for choosing the first alternative reduced to (*n*_*u*_ − *n*_*d*_) = 0 when $\mathbb {P}(U \in \mathcal {U}_{+}) = 0.97$. In this case, the decision-maker has such a strong prior (that asset values are rising) that it is optimal for them to choose the first alternative (buy an asset) even before making any observations.

**Fig. 9:**
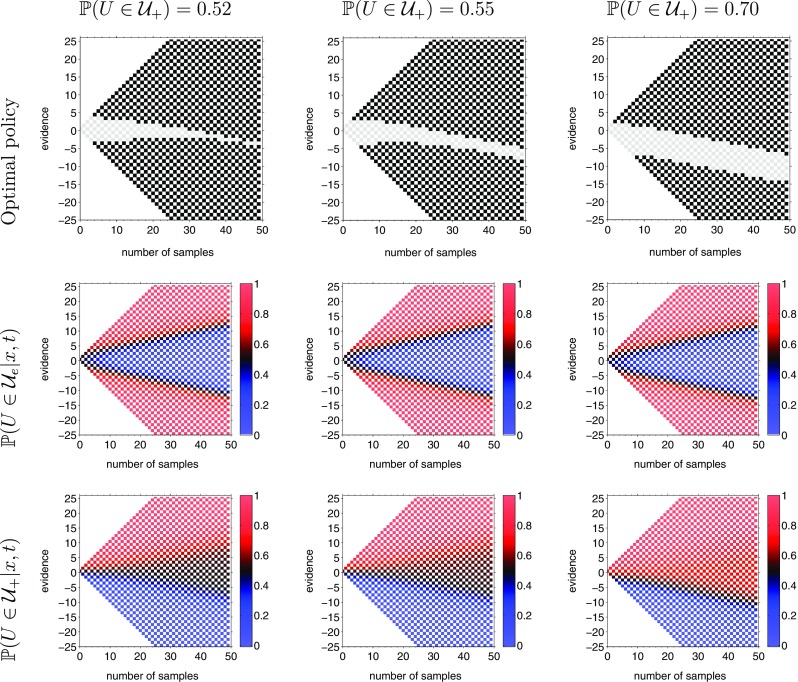
Optimal policy during mixed difficulty trials with biased prior beliefs. For all computations, the mixture of drifts involves *𝜖*_*e*_ = 0.20, *𝜖*_*d*_ = 0 and $\mathbb {P}(U \in \mathcal {U}_{e}) = \frac {1}{2}$. Three different priors are used: the left column uses $\mathbb {P}(U \in \mathcal {U}_{+}) = 0.52$, the middle column uses $\mathbb {P}(U \in \mathcal {U}_{+}) = 0.55$, and the right column uses $\mathbb {P}(U \in \mathcal {U}_{+}) = 0.70$. The *first row* shows optimal policies, the *second row* shows the posterior probability for the trial being easy given the state and the third row shows the posterior probability for the trial having *up-probability* > 0.50 given the state. For all three computations, the inter-trial intervals are *D*_*C*_ = *D*_*I*_ = 150

Thus, the optimal policy predicted by the above theory concurs with shifting the starting point when the task difficulty is fixed and known ahead of time. Let us now look at the mixed difficulty condition. Figure 9 shows the optimal policy for a mixed difficulty task with *up-probability* drawn from the set *u* ∈{0.30,0.50,0.70} and three different degrees of prior all biased towards the world being in the first state to varying degrees.

Like the single difficulty case, a prior bias that the world is more likely to be in the first state (asset values are more likely to rise) decreases the boundary for the first alternative (buy) and increases the boundary for the second alternative (sell). However, unlike the single difficulty case, this shift in boundaries is not constant, but changes with time: the optimal policies in Fig. 9 are *not* simply shifted along the evidence axis (compare with Fig. 8); rather, there are two components of the change in boundary. First, for all values of time, the distance to the upper boundary (for buy) is same-or-smaller than the equal-prior case (e.g., in the third column of Fig. 9, (*n*_*u*_ − *n*_*d*_) = 2 even at *t* = 2), and the distance to the lower boundary (for sell) is same-or-larger than the equal prior case. Second, the shift in boundaries is larger at longer durations (again most evident in the third column of Fig. 9, where it becomes optimal to choose the first alternative with the passage of time, even when the cumulative evidence is negative).

These optimal policies are in agreement with the dynamic prior model developed by Hanks et al. (2011), which proposes that the contribution of prior *increases* with time. To see this, consider the assets example again. Note that the prior used for generating the optimal policy in the third column of Fig. 9 corresponds to assets being more likely to rise than fall ($\mathbb {P}(U \in \mathcal {U}_{+}) = 0.70$). As time goes on, the cumulative evidence required to choose buy keeps decreasing while the cumulative evidence required to choose sell keeps increasing. In other words, with the passage of time, increasingly larger evidence is required to overcome the prior. This is consistent with a monotonically increasing dynamic prior signal proposed by Hanks et al. (2011).

The third column in Fig. 9 also shows another interesting aspect of optimal policies for unequal prior. In this case, the bias in prior is sufficiently strong and leads to a ‘time-varying region of indecision’: instead of the collapsing bounds observed for the equal-prior condition computations show optimal bounds that seem parallel *but* change monotonically with time.^5^So, for example, when $\mathbb {P}(U \in \mathcal {U}_{+})=0.70$, it is optimal for the decision-maker to keep waiting for more evidence even at large values of time, provided the current cumulative evidence lies in the grey (*wait*) region of the state-space.

The intuitive reason for this time-varying region of indecision is that, for states in this region, the decision-maker is neither able to infer if the trial is easy nor able to infer the true state of the world. To see this, we have plotted the posterior probability of the trial being easy in the second row of Fig. 9 and the posterior for the first state (rising) being the true state of the world in the third row. The posterior that the trial is easy does not depend on the prior about the state of the world: all three panels in second row are identical. However, the posterior on the true state of the world does depend on the prior beliefs: as the prior in favor of the world being in the first state (rising) increases, the region of intermediate posterior probabilities is shifted further down with the passage of time. The ‘region of indecision’ corresponds to an area of overlap in the second and third rows where the posterior probability that the trial is easy is close to 0.5 and the posterior probability that the true state of the world is rising is also close to 0.5 (both in black). Hence, the optimal thing to do is to *wait* and accumulate more evidence.

### Low confidence option

So far, we have considered two possible actions at every time step: to *wait* and accumulate more information, or to *go* and choose the more likely alternative. Of course this is not true in many realistic decision-making situations. For instance, in the example at the beginning of the article, the decision-maker may choose to examine the next asset in the portfolio without making a recommendation if they are unsure about their decision after making a sequence of *up*/*down* observations. We now show how the theory outlined above can be extended to such a case: the decision-maker has a third option (in addition to *wait* and *go*), which is to *pass* and move to the next trial with a reduced inter-trial delay. In this case, the MDP in Fig. 1(b) is changed to include a third option—*pass*—with a delay $D_{i}^{pass}$ but no immediate reward or penalty: $r_{ij}^{pass} = 0$. The policy iteration is carried out in the same way as above, except Eqs. 8 and 9 are updated to accommodate this alternative.

Kiani and Shadlen (2009) introduced an option similar to this *pass* action (they call it “opt-out”) in an experiment conducted on rhesus monkeys. The monkeys were trained to make saccadic eye movements to one of two targets that indicated the direction of motion of a set of moving dots on the screen (one of which was rewarded). In addition to being able to choose one of these targets, on a random half of the trials, the monkeys were presented a third saccadic target (a “sure target”) that gave a small but certain reward. This “opt-out” setting is similar to our extended model with a *pass* action with one distinction. Since Kiani and Shadlen (2009) did not use a fixed-time block paradigm where there was a trade-off between the speed and accuracy of decisions, they had to explicitly reward the “opt-out” action with a small reward. In contrast, we consider a setting where there is an implicit cost of time. Therefore it is sufficient to reduce the delay for the *pass* option without associating it with an explicit reward. Kiani and Shadlen (2009) found that the monkeys chose the sure target when their chance of making the correct decision about motion direction was small; that is, when the uncertainty of the motion direction was high.

**Fig. 10:**
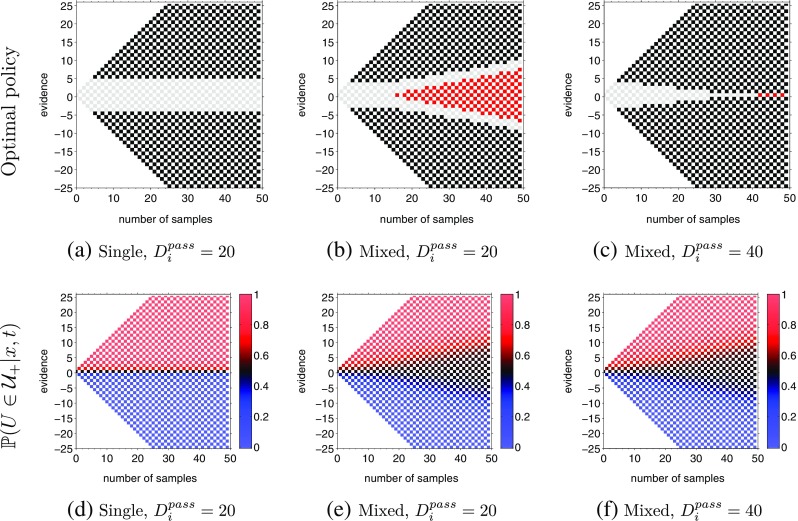
Optimal actions for all states when actions include the *pass* option. *Gray* = *wait*; *black* = *go*; *red* = *pass*. For all computations, *𝜖*_*e*_ = 0.20, *𝜖*_*d*_ = 0, *D*_*C*_ = *D*_*I*_ = 150. (**a**) The single difficulty case with $\mathbb {P}(U \in \mathcal {U}_{e}) = 1$. For (**b**) and (**c**), $\mathbb {P}(U \in \mathcal {U}_{e}) = \frac {1}{2}$. For (**a**) and (**b**), the inter-trial interval for *pass* action is 20 time steps while for (**c**) it is 40 time steps. Panels (**d**–**f**) show the corresponding posterior probabilities of a drift > 0.50, $\mathbb {P}(U \in \mathcal {U}_{+} | X_{t}=x)$, for the conditions in panels (**a**–**c**)

Figure 10 shows the optimal policy predicted by extending the above theory to include a *pass* option. For the single difficulty task (Fig. 10a), it is never optimal to choose the *pass* option. This is because choosing to pass has a cost associated with it (the inter-trial delay on passing) and no benefit—the next trial is just as difficult, so the same amount of information would need to be accumulated.

More interestingly, Fig. 10b and c show the optimal policy for the mixed difficulty task, with *up-probability* for each decision chosen from the set *u* ∈{0.30,0.50,0.70}. In agreement with the findings of Kiani and Shadlen (2009), the theory predicts that the *pass* action is a function of both evidence and time and is taken only in cases where the decision-maker has waited a relatively long duration and accumulated little or no evidence favoring either hypothesis. An inspection of the posterior probabilities, $\mathbb {P}(U \in \mathcal {U}_{+} | X_{t}=x)$, reveals why it becomes optimal to choose the *pass* option with the passage of time. It can be seen in Fig. 10e and f that for a fixed evidence *x*, as time increases, $\mathbb {P}(U \in \mathcal {U}_{+} | X_{t}=x)$ decreases (this is in contrast for the single difficulty case, Fig. 10d). Thus, with the increase in time, the confidence of the decision-maker in the same amount of cumulative evidence should diminish and the expected value of choosing the *pass* action becomes larger than the expected value of *wait* or *go*.

The computations also reveal how the optimal policies depend on the incentive provided for the pass option. In Fig. 10b, the inter-trial interval for the pass action is nearly an eighth of the interval for incorrect decisions while in Fig. 10c the inter-trial interval for the pass action is approximately a fourth of the interval for incorrect decision. Since all paths to the pass action is blocked by *go* action in Fig. 10c, the theory predicts that decreasing the incentive slightly should result in the optimal decision-maker never choosing the pass option.

## Discussion

### Key insights

Previous research has shown that when the goal of the decision-maker is to maximize their reward rate, it may be optimal for them to change their decision boundary with time. In this article, we have systematically outlined a dynamic programming procedure that can be used to compute *how* the decision boundary changes with time. Several important results were obtained by using this procedure to compute optimal policies under different conditions. Firstly, by removing the assumptions about deadlines of decisions and the cost of making observations, we found that neither of these were a pre-requisite for an optimal time-dependent decision boundary. Instead, what was critical, was a sequence of decisions with inter-mixed difficulties.

Next, by restricting the levels of difficulties to two, we were able to explore the effect of different difficulties on the shape of the decision boundaries. Our computations showed that optimal decision bounds do not necessarily decrease in the mixed difficulty condition and may, in fact, increase or remain constant. Computations using a variety of different difficulty levels revealed the following pattern: optimal bounds decreased when difficult trials (in mixed blocks) had lower optimal bounds than easy trials; they increased when the pattern was reversed, i.e., when difficult trials had higher optimal bounds than easy trials.

In addition to computing optimal boundaries, we also computed posterior probabilities for various inferences during the course of a decision. These computations provided insight into the reason for the shape of boundaries under different conditions. Optimal boundaries change with time only in the mixed difficulty condition and not in the single difficulty condition because observations made during the mixed difficulty condition provide the decision-maker two types of information: in addition to providing evidence about the true state of the world, observations also help the decision-maker infer the difficulty level of the current trial. At the start of the trial, the difficulty level of the current trial is determined by the decision-maker’s prior beliefs—e.g., that both easy and difficult trials are equally likely. So the optimal decision-maker starts with decision boundaries that reflect these prior beliefs. As the trial progresses, the decision-maker uses the cumulative evidence *as well as* the time spent to gather this evidence to update the posterior on the difficulty level of the trial. They use this posterior to then update the decision boundary dynamically. In cases where the decision boundary for the difficult trials is lower (higher) than easy trials, the decision-maker can maximize their reward rate by decreasing (increasing) the decision boundary with time.

Similarly, the model also provided insight into the relationship between the shape of optimal decision boundaries and priors on the state of the world. When priors are unequal, observations in mixed difficulty trials provide three types of information. They can be used to perform the two inferences mentioned above—about the true state of the world and the difficulty of the trial—but additionally, they can also be used to compute the weight of the prior. Computations showed that it is optimal for the decision-maker to increase the weight of the prior with time, when decisions have inter-mixed difficulties and the decision-maker has unequal priors. A possible explanation for this counter-intuitive finding is that the optimal decision-maker should consider the *reliability* of signals when calculating how much weight to give the prior. As the number of observations increase, the reliability of the evidence decreases and the optimal decision-maker should give more weight to the prior. Note that this is the premise on which (Hanks et al. 2011) base their “dynamic bias” model. Our computations show how the dynamic bias signal should change with time when the goal of the decision-maker is to maximize the reward rate.

### Implications for empirical research

Using the dynamic programming procedure to predict optimal policies provides a strong set of constraints for observing different boundary shapes. In particular, we found that optimal boundaries decreased appreciably under a limited set of conditions and only if one type of decision is extremely difficult. This observation is particularly relevant to a number of recent studies that have investigated the shape of decision boundaries adopted by participants in decision-making experiments.

Hawkins et al. (2015) performed a meta-analysis of reaction-time and error-rate data from eight studies using a variety of different paradigms and found that, overall, these data favored a fixed bounds model over collapsing bounds models in humans. Similarly, (Voskuilen et al., 2016) carried out a meta-analysis using data from four numerosity discrimination experiments and two motion-discrimination experiments and found that data in five out of six experiments favored fixed boundaries over collapsing boundaries.

The majority of experiments included in these meta-analyses consider mixed difficulty blocks with a larger number of difficulty levels than we have considered so far. For example, one of the studies considered by both Hawkins et al. (2015) and Voskuilen et al. (2016) is Experiment 1 from (Ratcliff and McKoon, 2008), who use a motion-discrimination task with motion coherence that varies from trial to trial across six different levels (5%,10%,15%,25%,35%,50%). It is unclear what the shape of optimal bounds should be for this mixture of difficulties, especially because we do not know what participants were trying to optimize in this study. However, even if participants were maximizing reward rate, we do not know whether they should decrease their decision boundaries under these conditions.

It is possible to extend the above framework to more than two difficulties and make predictions about the shape of optimal boundaries in such settings. However, one problem is that this framework assumes an exact knowledge of different drifts, *𝜖*, used in the mixture of difficulties. In the experiments considered by Hawkins et al. (2015) and Voskuilen et al. (2016), we do not know the exact values these drifts take since the paradigms used in these studies (motion coherence, numerosity judgment, etc.) involve implicit sampling of evidence.

One advantage of the *expanded judgment paradigm* is that the experimenter is able to directly observe the drift of samples shown to the participant and compare the decision boundary used by participants with the one predicted by reward-rate maximization. We have recently conducted a large series of experiments in which we adopted this approach, adopting a very explicit reward structure and creating conditions for which the model predicts that boundaries should change when different difficulty levels are mixed (Malhotra, Leslie, Ludwig, & Bogacz, in press).^6^ We found that participants indeed modulated the slope of their decision boundaries in the direction predicted by maximization of reward rate.

In order to understand why our findings contrast with those of Hawkins et al. (2015) and Voskuilen et al. (2016), we extended the model to accommodate any number of difficulty levels. Instead of assuming that the *up-probability* comes from the set ${\mathcal {U}_{e} \cup \mathcal {U}_{d}}$, we assumed that $u \in \mathcal {U}$, where $\mathcal {U}$ is a set of *up-probabilities* with *n* different drifts, {*𝜖*_1_,…, *𝜖*_*n*_}. We then attempted to model the set of studies analyzed by Hawkins et al. (2015) and Voskuilen et al. (2016).

As mentioned above, one problem is that we do not know what the actual drift of the evidence was in these studies. Our strategy was to match the observed error rates for a set of difficulties in the original experiment with the error rates for the optimal bounds predicted by a corresponding set of drifts (see Appendix B for details). We found that a range of reasonable mappings between the difficulties used in an experiment and the set of drifts {*𝜖*_1_,…, *𝜖*_*n*_} gave fairly similar shapes of optimal boundaries.

Another problem is that it is unclear how the inter-trial interval used in the experiments maps on to the inter-trial interval used in the dynamic programming procedure. More precisely, in the dynamic programming procedure the inter-trial interval is specified as a multiple of the rate at which the evidence is delivered. However, due to the implicit sampling in the original experiment, we do not know the relation between the (internal) evidence sampling rate and the inter-trial intervals. Therefore, we computed the optimal policies for a wide range of different inter-trial intervals. As we show below, even though the optimal policy changes with a change in inter-trial interval, the slope of the resulting optimal decision boundaries remain fairly similar across a wide range of intervals.

Table 1 summarizes the conditions used in experiments, the distribution of these conditions across trials and a corresponding set of drifts used in the dynamic programming procedure. We also matched the distribution of difficulty levels used in these experiments with the distribution of drifts used for our computations. We chose this set of experiments so that they cover the entire range of mixture of difficulties considered across experiments considered by Hawkins et al. (2015) and Voskuilen et al. (2016).

**Table 1:** Set of studies and drifts used to generate optimal policies

Study	Paradigm	Conditions	Distribution	Drifts ({*𝜖*_1_…*𝜖*_*n*_})
PHS_05	Motion	$\left \{0\%, 3.2\%, 6.4\%,\right .$	Uniform	$\left \{0, 0.03, 0.05,\right .$
		$\left . 12.8\%, 25.6\%, 51.2\%\right \}$		$\left . 0.10, 0.20, 0.40\right \}$
RTM_01	Distance	32 values	Uniform	17 values
		Range: [1.7, 2.4] cm		Range: [0, 0.50]
R_07	Brightness	$\left \{\text {Bright}: 2\%, 35\%, 45\%,\right .$	Uniform	{0.05, 0.10, 0.20}
		$\left . \text {Dark:} 55\%, 65\%, 98\%\right \}$		
RM_08	Motion	$\left \{5\%, 10\%, 15\%,\right .$	Uniform	$\left \{ 0.04, 0.07, 0.10 \right .$
		$\left . 25\%, 35\%, 50\%\right \}$		$\left . 0.15, 0.20, 0.30\right \}$
MS_14	Color	$\left \{35\%, 42\%, 46\%, \right .$	Uniform	{0, 0.05, 0.10, 0.20}
		$\left . 50\%, 54\%, 58\%, 65\%\right \}$		
VRS_16: E1	Numerosity	Range: [21, 80]	Piecewise	$\left \{0, 0.02, 0.04, 0.06 \right .$
			Uniform	$\left . 0.30, 0.32, 0.34, 0.36\right \}$
VRS_16: E2	Numerosity	Range: [3, 98]	Approximately	{0, 0.05,…, 0.50}
			Gaussian	
VRS_16: E3	Numerosity	Range: [31, 70]	Uniform	{0, 0.02,…, 0.20}
VRS_16: E4	Numerosity	Range: [3, 98]	Uniform	{0, 0.02,…, 0.48}

*Notes.* Each row shows the set of conditions used in the experiment, the distribution of these conditions across trials and the set of drift parameters used to compute optimal policies in Fig. 11. The value given to a condition refers to the motion coherence for the motion discrimination task, to the separation of dots for the distance judgment task, to the proportion of black pixels for the brightness discrimination task, to the percentage of cyan to magenta checkers for the color judgment task and to the number of asterisks for the numerosity judgment task. For the computation VRS_16: E2, the probability of each drift value *𝜖* was equal to $\frac {1}{Z} \mathcal {N}(\epsilon ; \mu , \sigma )$, where $\mathcal {N}(\cdot )$ is the probability density of the normal distribution with *μ* = 0, and standard deviation *σ* = 0.21 and *Z* is a normalization factor ensuring that the probabilities add up to 1. The names of studies are abbreviated as follows PHS_05: (Palmer, Huk, & Shadlen, 2005), RTM_01: (Ratcliff, Thapar, & McKoon, 2001), R_07: (Ratcliff, Hasegawa, Hasegawa, Smith, & Segraves, 2007), RM_08: (Ratcliff & McKoon, 2008), MS_14: (Middlebrooks & Schall, 2014), VRS_16: (Voskuilen et al., 2016) with E1 …E4 standing for Experiments 1 … 4, respectively.

**Fig. 11:**
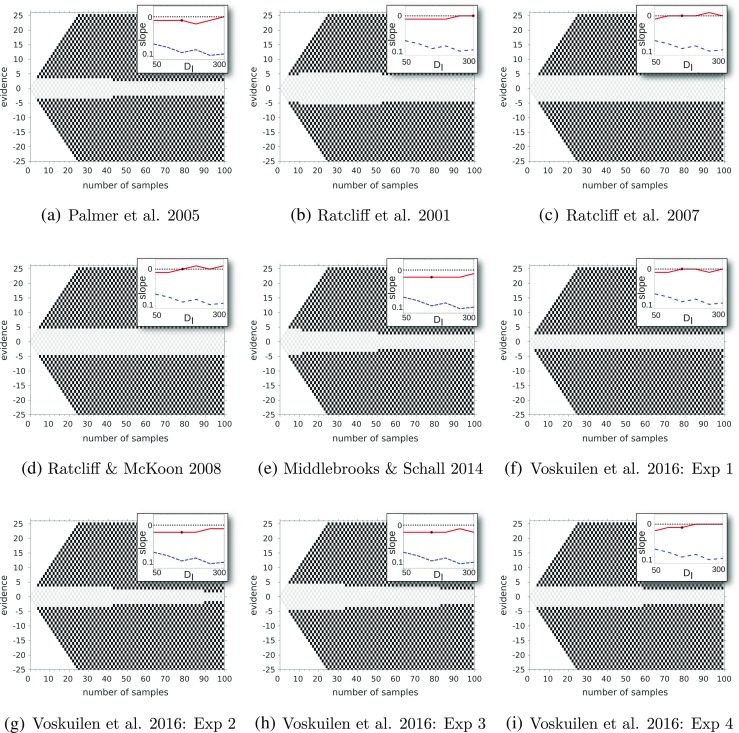
Optimal policies for mixture of difficulties used in experiments considered by Hawkins et al. (2015) and Voskuilen et al. (2016). *Insets* show the slope of the optimal boundary (measured as tangent of a line fitting the boundary) across a range of inter-trial intervals for the mixture of drifts that maps to the experiment (*solid, red line*) and compares it to flat boundaries (*dotted, black line*) and the mixture *𝜖* ∈{0.20,0.50}, which gives a large slope across the range of inter-trial intervals (*dashed, blue line*). The *dot* in the inset along each solid (*red*) line indicates the value of inter-trial interval used to generate the optimal policy shown in the main figure

Figure 11 shows the optimal policies obtained by using the dynamic programming procedure for each mixture of drifts in Table 1. While the shape of any optimal boundary depends on the inter-trial interval (as discussed above), we found that the slope of optimal boundaries remained similar for a range of different inter-trial intervals and the inset in each figure shows how this slope changes with a change in inter-trial interval. The insets also compare this slope (solid, red line) with the flat boundary (dotted, black line) and the optimal slope for a mixture of two difficulties, *𝜖* ∈{0,0.20}, which leads to rapidly decreasing bounds (dashed, blue line). A (red) dot in each inset indicates the value of inter-trial interval used to plot the policies in the main plot. All policies have been plotted for the same value of inter-trial interval used in the computations above, i.e., *D*_*C*_ = *D*_*I*_ = 150, except Fig. 11b, which uses *D*_*C*_ = *D*_*I*_ = 300 to highlight a property of optimal policies observed when a task consists of more than two difficulty levels (see below).

Extending the framework to more than two difficulties reveals two important results. First, the optimal bounds are nearly flat across the range of mixed difficulty tasks used in these experiments. In order to monitor the long-term trend for these slopes, we have plotted each of the policies in Fig. 11 till time step *t* = 100 (in contrast to *t* = 50 above). In spite of this, we observed very little change in optimal evidence as a function of number of samples observed during a trial. Optimal bounds do seem to decrease slightly for some mixed difficulty tasks when they include a substantial proportion of “very difficult” trials (e.g., MS_14 and VRS_16: E2, E3). However, even in these cases, the amount of decrease is small (compare the solid, red line to the dashed, blue line which corresponds to a mixture that gives a large decrease) and it would be very difficult to distinguish between constant or decreasing bounds based on the reaction-time distributions obtained from these bounds. Second, for some mixtures of difficulties, the optimal bounds are a non-monotonic function of time where the optimal boundary first increases, then remains constant for some time and finally decreases (see, for example, Fig. 11b). This non-monotonic pattern occurred only when number of trial difficulties was greater than two.

Clearly these computations and in particular their mapping onto the original studies have to be interpreted with caution, due to the difficulties in translating continuous, implicit sampling paradigms to the discrete expanded judgment framework. Nevertheless, our wide-ranging exploration of the parameter space (mixtures of difficulties and inter-trial intervals) suggests that the optimal boundaries in these experiments may be very close to flat boundaries. In that case, even if participants maximized reward rate in these experiments, it may be difficult to identify subtly decreasing boundaries on the basis of realistic empirical evidence. Of course, we do not know what variable participants were trying to optimize in these studies. The computations above highlight just how crucial an explicit reward structure is in that regard.

### Reward-rate maximization

A related point is that many decision-making tasks (included those considered in the meta-analyses mentioned above) do not carefully control the reward structure of the experiment. Many studies instruct the participant simply to be “as fast and accurate as possible”. The model we consider in this study is unable to make predictions about the optimal shape of boundaries in these tasks, because it is not clear what the participant is optimizing. It could be that when the goal of the participant is not precisely related to their performance, they adopt a strategy such as “favor accuracy over speed” or “minimize the time spent in the experiment and leave as quickly as possible, without committing a socially unacceptable proportion of errors” (Hawkins, Brown, Steyvers, & Wagenmakers, 2012). On the other hand, it could also be that when people are given instructions that precisely relate their performance to reward, the cost required to estimate the optimal strategy is too high and people simply adopt a heuristic – a fixed threshold – that does a reasonable job during the task.

More generally, one could question whether people indeed try to maximize reward rate while making sequential decisions and hence the relevance of policies that maximize reward rate for empirical research. After all, a number of studies have found that people tend to overvalue accuracy and set decision boundaries that are wider than warranted by maximizing reward rate (Maddox and Bohil, 1998; Bohil & Maddox, 2003; Myung & Busemeyer, 1989), especially with an increase in the difficulty of trials (Balci et al., 2011; Starns and Ratcliff, 2012) and with the increase in speed of the decision-making task (Simen et al., 2009). To explain this behavior, a set of studies have investigated alternative objective functions (Bohil & Maddox, 2003; Bogacz et al., 2006; Zacksenhouse et al., 2010). For example, Bogacz, Hu, Holmes and Cohen, 2010) found that only about 30% of participants set the boundaries to the level maximizing reward rate. In contrast, the bounds set by the majority of participants could be better explained by maximization of a modified reward rate which includes an additional penalty (in a form of negative reward) after each incorrect trial, although no such penalty was given in the actual experiment (Bogacz et al., 2010). Analogous virtual penalties for errors imposed by the participants themselves can be easily incorporated in the proposed framework by making *R*_*I*_ more negative (Eq. 8).

However, understanding the behavior that maximizes reward rate is important for several reasons. Firstly, recent evidence indicates that the decision boundaries adopted by human participants approach reward-rate optimizing boundaries, in single difficulty tasks, provided participants get enough training and feedback (Evans & Brown, 2016). This suggests that people use reward rate to learn the decision boundaries over a sequence of trials.

Secondly, the shape of the reward landscape may explain why people adopt more cautious strategies than warranted by maximizing reward rate. In a recent set of experiments, we used an expanded judgment task to directly infer the decision boundaries adopted by participants and found that participants may be choosing decision boundaries that trade off between maximizing reward rate and the cost of errors in the boundary setting (Malhotra et al., in press). That is, we considered participants’ decision boundaries on a “reward landscape” that specifies how reward rate varies as a function of the height and slope of the decision boundary. We noted that these landscapes were asymmetrical around the maximum reward rate, so that an error in the “wrong” direction would incur a large cost. Participants were generally biased away from this “cliff edge” in the reward landscape. Importantly, across a range of experiments, participants were sensitive to experimental manipulations that modified this global reward landscape. That is, participants shifted their decision boundaries in the direction predicted by the optimal policies shown in Fig. 3 when the task switched from single to mixed difficulties. This happened even when the task was fast-paced and participants were given only a small amount of training on each task. Thus, even though people may not be able to maximize reward rate, they are clearly sensitive to reward-rate manipulations and respond adaptively to such manipulations by changing their decision boundaries.

Lastly, the optimal policies predicted by the dynamic programming procedure above provides a normative target for the (learned or evolved) mechanism used by people to make decisions. Thus, these normative models provide a framework for understanding empirical behavior; if people deviate systematically from these optimal decisions, it will be insightful to understand why, and under what conditions, they deviate from a policy that maximizes the potential reward and how these alternative objective functions relate to reward-rate maximization.

### Assumptions and generalizations

We have made a number of assumptions in this study with the specific aim of establishing the minimal conditions for time-varying decision boundaries and exploring how properties of decision (such as difficulty) affects the shape of decision bounds.

Firstly, note that in contrast to previous accounts that use dynamic programming to establish optimal decision boundaries (e.g., Drugowitsch et al., 2012; Huang & Rao, 2013), we compute optimal policies directly in terms of evidence and time, rather than (posterior) belief and time. There are two motivations for doing this. Firstly, our key goal here is to understand the shape of optimal decision boundaries for sequential sampling models which define boundaries in terms of evidence. Indeed, most studies which have aimed to test whether decision boundaries collapse, do so by fitting sequential sampling or accumulator models to reaction time and error data (Ditterich, 2006; Drugowitsch et al., 2012; Hawkins et al., 2015; Voskuilen et al., 2016). Secondly, we do not want to assume that the decision-making system necessarily computes posterior beliefs. This means that the decision-making process that aims to maximize reward rate can be implemented by a physical system integrating sensory input. For an alternative approach, see (Drugowitsch et al., 2012), who use dynamic programming to compute the optimal boundaries in belief space and then map these boundaries to evidence space.

Next, a key assumption is that policies can be compared on the basis of reward rate. While reward rate is a sensible scale for comparing policies, it may not always be the ecologically rational measure. In situations where the number of observations are limited (e.g., Rapoport & Burkheimer, 1971; Lee & Zhang, 2012) or the time available for making a decision is limited (e.g., Frazier & Yu, 2007), the decision-maker should maximize the expected future reward rather than the reward rate. If the number of decisions are fixed and time is not a commodity, then the decision-maker should maximize the accuracy. In general, the ecological situation or the experiment’s design will determine the scale on which policies can be compared.

Another simplifying assumption is that the decision-maker’s environment remains stationary over time. In more ecologically plausible situations, parameters such as the drift rate, inter-stimulus interval and reward per decision will vary over time. For example, the environment may switch from being plentiful (high expectation of reward) to sparse (low expectation of reward). In these situations, each trial will inform the decision-maker about the state of the environment and the normative decision-maker should adapt the boundary from trial-to-trial based on the inferred state. Such an adaptive model was first examined by Vickers (1979), who proposed a confidence-based adjustment of decision-boundary from trial-to-trial. Similarly, (Simen, Cohen, & Holmes, 2006) proposed a neural network model that continuously adjusts the boundary based on an estimate of the current reward rate.

Recent experiments have shown that participants indeed respond to environmental change by adapting the gain of each stimulus (Cheadle et al., 2014) or the total amount of evidence examined for each decision (Lee, Newell, & Vandekerckhove, 2014). Lee et al. (2014) found that sequential sampling models can capture participant behavior in such environments by incorporating a regulatory mechanism like confidence, i.e. a confidence-based adjustment of decision boundary. However, they also found large individual differences in the best-fitting model and in the parameters chosen for the regulatory mechanism. An approach that combines mechanistic models such as those examined by Lee et al. (2014) and normative models such as the one discussed above could explain why these individual differences occur and how strategies differ with respect to a common currency, such as the average reward.

## Figures and Tables

**Fig. 12:**
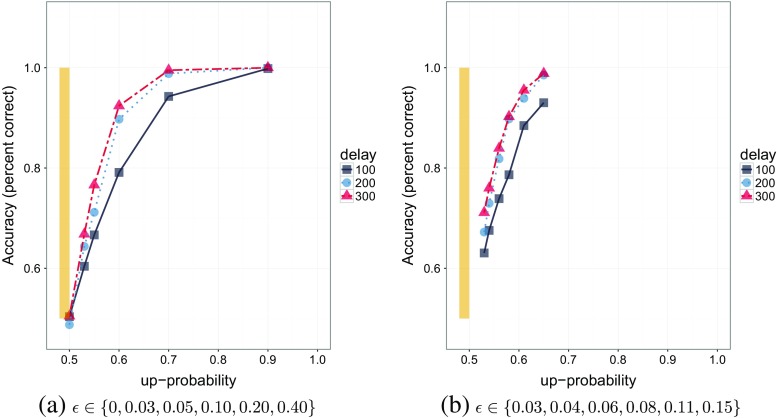
A comparison of accuracies between decisions simulated from optimal bounds and decision performed by participants. The two panels show the accuracy of 10,000 simulated decisions during two mixed difficulty tasks, each of which use a mixture of six difficulty levels but differ in the range of difficulties. Panel (**a**) uses a large range of drifts [0,0.40], while panel (**b**) uses a comparatively smaller range [0.03,0.15]. *Squares*, *circles*, and *triangles* show these accuracies for an inter-trial delay, *D*_*I*_, of 100, 200, and 300 time units, respectively. The *yellow bar* on the left of each panel shows the range of accuracies observed in Experiment 1 of Palmer et al. (2005) which used six different motion coherence levels: {0%, 3.2%, 6.4%, 12.8%, 25.6%, 51.2%}

## Appendix A: Eventually it is optimal to go at zero

In this appendix we show that, under a mild condition, it will be optimal to guess a hypothesis when the evidence level *x* is 0 for sufficiently large *t*. In other words, the bounds do eventually collapse to 0. The situation we envisage is one in which some of the trials in the mixed condition have zero drift, i.e., *𝜖*_*d*_ = 0, so that

$$\begin{array}{@{}rcl@{}} p_{0}:=\mathbb{P}\left( U\,=\,\frac12\right) \!>\! 0,\! \quad \!\mathbb{P}\left( U\,=\,\frac12+\epsilon_{e}\right) \,=\, \mathbb{P}\left( U\!=\frac12-\epsilon_{e}\right) \,=\, \frac{1-p_{0}}2. \end{array} $$

We also assume that the decision-maker gets a unit reward for making correct decisions and no reward for making incorrect decisions and there is a fixed inter-trial delay *D* between taking a *go* action and returning to the zero-value state (0,0).

We start with some observations. First note that the decision-maker could use a policy which always guesses at *t* = 0. This policy scores on average $\frac 12$ per trial, and trials take *D* time units (since there is no time spent gathering evidence). Hence the average reward per unit time of this guessing policy is $\frac 1{2D}$. The optimal policy $\hat \pi $ will therefore have average reward per unit time, $\rho ^{\hat {\pi }}\geq \frac 1{2D}$. Similarly, an oracle policy can guess correctly at time 0, and achieve an average reward per unit time of $\frac 1D$; since the performance of any policy is bounded above by this perfect instant guessing policy, $\rho ^{\hat {\pi }}\leq \frac 1D$. We have shown that

10 $$ \frac{1}{2D} \leq \rho^{\hat{\pi}} \leq \frac1D. $$

Along similar lines, and recalling that we have fixed $v^{\pi }_{(0,0)}=0$, note that the maximum possible reward resulting from choosing a hypothesis is equal to 1, and there will be delay at least *D* in transitioning from (*t*, *x*) to (0,0), so for any *π*, *x* and *t*

11 $$ v^{{\pi}}_{(t,x)} \leq 1-\rho^{\pi} D. $$

We now prove that for a sufficiently large *T*, the optimal action in the state (*T*, 0) is *go*. This will be true if the value of taking the action *go*, in state (*T*, 0), is larger than taking the action *wait*. If we denote the value of taking action *a* in state (*t*, *x*) under policy *π* by $Q_{(t,x)}^{\pi }(a)$, we can write this condition as $Q_{(T,0)}^{\pi }(go) > Q_{(T,0)}^{\pi }(wait)$. Note that $Q_{(T,0)}^{\pi }(go) = \frac 12-\rho ^{\pi } D$, since the selected hypothesis is correct with probability $\frac 12$ and then there is a delay of *D* before returning to the zero-value state (0,0). Therefore, we would like to prove that $Q_{(T,0)}^{\pi }(wait) < \frac 12-\rho ^{\pi } D$.

Now, consider a time window of duration Δ after *T*. The value of waiting at (*T*, 0) will depend on one of two future outcomes during this time window. Either:

- the decision-maker will choose the action *go* after *τ* < Δ steps, achieving a value of $Q^{\pi }_{(T+\tau ,x)}(go)$ and incurring an additional waiting cost of *ρ* ^*π*^ *τ*, or
- the decision-maker will still be waiting until time *T* + Δ, achieving value $v_{(t+{\Delta },x)}^{\pi }$ but incurring an additional waiting cost of *ρ* ^*π*^Δ.

Therefore the value of *wait*ing at (*T*, 0) is a convex combination of the time-penalized value of these future outcomes, so

12 $$ Q_{(T,0)}^{\pi}(wait) \!\leq\! \max\!\left\{\!\max_{1\leq\tau<{\Delta},|x|\leq \tau} \{Q^{\pi}_{(T+\tau,x)}(go)\,-\,\rho^{\pi}\tau\}, v_{(t+{\Delta},x)}^{\pi}\,-\,\rho^{\pi}{\Delta}\!\right\}. $$

Note that by Eq. 11, $v_{(t+{\Delta },x)}^{\pi } \leq 1-\rho ^{\pi } D$. Also note that $Q^{\pi }_{(T+\tau ,x)}(go)$ is the expected instantaneous reward from the action, plus a time penalty of *ρ* ^*π*^ *D*. We will show below that, for any *η* > 0, we can choose *T* sufficiently large that this expected instantaneous reward is less than or equal to $\frac 12+\eta $. Therefore $Q^{\pi }_{(T+\tau ,x)}(go) \leq \frac 12+\eta -\rho ^{\pi }D$. Hence

$$Q_{(T,0)}^{\pi}(wait) \!\leq\! \max\!\left\{\max_{1\leq\tau<{\Delta}} \{\frac12\,+\,\eta\,-\,\rho^{\pi}(\tau\,+\,D)\}, \!1\,-\,\rho^{\pi}D\,-\,\rho^{\pi}{\Delta}\right\}. $$

For an interval Δ = 2*D*

$$Q_{(T,0)}^{\pi}(wait) \leq \max\left\{\frac12+\eta-\rho^{\pi}, 1-2\rho^{\pi}D\right\}-\rho^{\pi}D. $$

Now 1 − 2*ρ* ^*π*^ *D* ≤ 0 by Eq. 10 and if we choose *η* (which is an arbitrary constant) to be such that $\eta <\frac 1{2D}$, then *η* < *ρ* ^*π*^ and it follows that

$$Q_{(T,0)}^{\pi}(wait) < \frac12 - \rho^{\pi}D = Q_{(T,0)}^{\pi}(go). $$

The optimal action at (*T*, 0) is therefore to *go*.

It remains to show that, if *T* is sufficiently large, the expected instantaneous reward is bounded by $\frac 12 + \eta $. The expected instantaneous reward in any state is equal to the size of the reward times the probability of receiving it. Since we assume that the reward size is one unit and decision-makers receive a reward only for correct decisions, the expected instantaneous reward in a state (*t*, *x*) is $p_{(t,x) \rightarrow C}^{go}$. From Eq. 7, we know that

$$p_{(t,x) \rightarrow C}^{go} \;\,=\, \; max \left\{\mathbb{P}(U \!\in \mathcal{U}_{+} | X_{t}\,=\,x), \mathbb{P}(U \!\in \mathcal{U}_{-} | X_{t}\,=\,x)\right\}, $$

and in the special case where *𝜖*_*d*_ = 0, $\mathbb {P}(U \in \mathcal {U}_{+} | X_{t}=x)$ can be replaced by $\mathbb {P}(U = \frac {1}{2}+\epsilon _{e} | X_{t}=x) + \frac {1}{2} \mathbb {P}(U=\frac {1}{2} | X_{t}=x)$. Recall that each of the paths that reach *X*_*t*_ = *x* contain $n_{u} = \frac {t+x}{2}$ upward transitions and $n_{d} = \frac {t-x}{2}$ downward transitions. From Eq. 5 we have that

$$\mathbb{P}(U=u\,|\, X_{t}=x) = \frac{u^{n_{u}}(1-u)^{n_{d}} \mathbb{P}(U=u)} {{\sum}_{\tilde{u}\in\mathcal{U}}{\tilde{u}}^{n_{u}}(1-{\tilde{u}})^{n_{d}} \mathbb{P}(U=\tilde{u})}. $$

Hence by Eq. 7 the expected instantaneous reward under the action *go* when *x* ≥ 0 (and the hypothesis + is selected) is therefore

$$\begin{array}{@{}rcl@{}} p_{(t,x) \rightarrow C}^{go} &=& \mathbb{P}\left( U = \frac{1}{2}+\epsilon_{e} | X_{t}=x\right) + \frac{1}{2} \mathbb{P}\left( U=\frac{1}{2} | X_{t}=x\right)\\ &=& \frac{\left( \frac12+\epsilon\right)^{n_{u}} \left( \frac12-\epsilon\right)^{n_{d}} \frac{1-p_{0}}2 + \frac12 \left( \frac12\right)^{n_{u}} \left( \frac12\right)^{n_{d}} p_{0}} {\left( \frac12+\epsilon\right)^{n_{u}} \left( \frac12-\epsilon\right)^{n_{d}} \frac{1-p_{0}}2 + \left( \frac12\right)^{n_{u}} \left( \frac12\right)^{n_{d}} p_{0} + \left( \frac12-\epsilon\right)^{n_{u}} \left( \frac12+\epsilon\right)^{n_{d}} \frac{1-p_{0}}2}\\ &=& \frac{(1-4\epsilon^{2})^{\frac{t-x}2} (1+2\epsilon)^{x}(1-p_{0}) + p_{0}} {(1-4\epsilon^{2})^{\frac{t-x}2} [(1+2\epsilon)^{x}+(1-2\epsilon)^{x}] (1-p_{0}) + 2p_{0}}. \end{array} $$

For fixed *x*, $ (1-4\epsilon ^{2})^{\frac {t-x}2}\to 0$ as $t\to \infty $, so that the expected reward from going at *x* converges to $\frac 12$ as *t* becomes large. Since the maximization in Eq. 12 is over |*x*|≤ *τ* < Δ, we can take *T* sufficiently large that the expected instantaneous reward from the action *go* in any state (*T* + *τ*, *x*) with 1 ≤ *τ* < Δ and |*x*|≤ *τ* is less than $\frac 12 + \eta $. So for any *η* we can say the following: for any *x*, for a sufficiently large *t* ≥ *T*, the instantaneous reward for *go* ing at (*t*, *x*) is less than $\frac 12+\eta $. An identical calculation holds for *x* ≤ 0.

## Appendix B: Mapping experimental conditions to drifts

We now describe how we selected a set of drifts corresponding to each study in Table 1. Since these studies do not use an expanded judgment paradigm, we do not explicitly know the values of drift parameter; instead, these studies specify a measurable property of the stimulus, such as motion coherence, that is assumed to correlate with the drift. However, we know how the participants performed in this task – i.e. their accuracy – during each of these coherence conditions. These accuracy levels constrain the possible range of drift rates that correspond to the motion coherences and can be used to infer an appropriate range of drifts.

Specifically, we used the following method to determine whether any given set of drift rates, {*𝜖*_1_,…, *𝜖*_*n*_}, approximated the conditions for a study: (i) we used the dynamic programming procedure described in the main text to compute the optimal bounds for a mixed difficulty task with difficulties drawn from the given set of drifts and for a range of inter-trial delays, *D*_*I*_; (ii) we simulated decisions by integrating noisy evidence to these optimal bounds, with the drift rate of each trial chosen randomly from the given set of drifts; (iii) we determined the accuracy levels, *a*_1_,…, *a*_*n*_, of these simulated decisions; (iv) finally, we compared the accuracy levels in the original study with the accuracy levels for each drift for the simulated decisions. We rejected any given set of drifts that underestimated or overestimated the empirically observed range of accuracies for the chosen range of inter-trial delays. This left us with a set of drifts, shown in Table 1, that approximately matched the level of accuracies in the original study.

For example, Fig. 12 shows the accuracies for decisions simulated in the above manner by computing optimal bounds for two different sets of drifts: {0, 0.03, 0.05, 0.10, 0.20, 0.40} and {0.03, 0.04, 0.06, 0.08, 0.11, 0.15}. Each mixture contains six different difficulties, just like the original study conducted by Palmer et al. (2005). We performed these simulations for a range of inter-trial delays and Fig. 12 shows three such delays. The (yellow) bar on the left of each panel shows the empirically observed range of accuracies. It is clear that the range of difficulties in Fig. 12b considerably underestimates the empirically observed range of accuracies and therefore is not an appropriate approximation of the difficulties used in the original study. On the other hand, the range of difficulties in Fig. 12a captures the observed range of accuracies for a variety of inter-trial delays.

Figure 12 also illustrates that the mapping between drift rates and error rates is complex since the parameter space is highly multi-dimensional—with accuracy a function of the inter-trial delay as well as the *n* values in the set {*𝜖*_1_…*𝜖*_*n*_}. In order to choose an appropriate mapping, we explored a considerable range of delays and sets of drifts. While the complexity of this parameter space makes it difficult to be absolutely certain that there is no combination of a set of drifts and delays for which more strongly decreasing boundaries are seen, our observation was that the optimal boundaries shown in Fig. 11 were fairly typical of each study for reasonable choice of parameters. Where more strongly decreasing boundaries were seen, they were (a) still much shallower than the optimal boundaries for the mixture of two difficulties (*𝜖* ∈{0, 0.20}, as conveyed in insets for Fig. 11), and (b) the predicted accuracy levels did not match those that were empirically observed.

## Footnotes

1 Throughout this article, we use ‘threshold’ to refer to a decision boundary that remains constant within and across trials.

2 We will revise this assumption in the section titled “Extensions of the model” below.

3 We assume all policies considered will eventually *go*, so that the system is ergodic and the limiting state probabilities are independent of the starting state.

4 The sawtooth (zigzag) pattern in Fig. 5(d–f) is a consequence of the discretization of time and evidence. For example, moving from left to right along the boundary in Fig. 5d, the value of evidence oscillates between |*n*_*u*_ − *n*_*d*_| = 6 and |*n*_*u*_ − *n*_*d*_| = 7, leading to the oscillation in the value of the posterior probability $\mathbb {P}(U \in \mathcal {U}_{+} | X_{t} = x)$.

5 This pattern seemed to hold even when we increased *t*_*max*_ to 100. Further research would be required to investigate if this is analytically the case when $t_{max} \rightarrow \infty $.

6 The authors’ pre-print accepted for publication is available at https://osf.io/2rdrw/.
